# Ablation of Arginylation in the Mouse N-End Rule Pathway: Loss of Fat, Higher Metabolic Rate, Damaged Spermatogenesis, and Neurological Perturbations

**DOI:** 10.1371/journal.pone.0007757

**Published:** 2009-11-13

**Authors:** Christopher S. Brower, Alexander Varshavsky

**Affiliations:** Division of Biology, California Institute of Technology, Pasadena, California, United States of America; New Mexico State University, United States of America

## Abstract

In the N-end rule pathway of protein degradation, the destabilizing activity of N-terminal Asp, Glu or (oxidized) Cys residues requires their conjugation to Arg, which is recognized directly by pathway's ubiquitin ligases. N-terminal arginylation is mediated by the Ate1 arginyltransferase, whose physiological substrates include the Rgs4, Rgs5 and Rgs16 regulators of G proteins. Here, we employed the Cre-lox technique to uncover new physiological functions of N-terminal arginylation in adult mice. We show that postnatal deletion of mouse *Ate1* (its unconditional deletion is embryonic lethal) causes a rapid decrease of body weight and results in early death of ∼15% of Ate1-deficient mice. Despite being hyperphagic, the surviving Ate1-deficient mice contain little visceral fat. They also exhibit an increased metabolic rate, ectopic induction of the Ucp1 uncoupling protein in white fat, and are resistant to diet-induced obesity. In addition, Ate1-deficient mice have enlarged brains, an enhanced startle response, are strikingly hyperkinetic, and are prone to seizures and kyphosis. Ate1-deficient males are also infertile, owing to defects in *Ate1^−/−^* spermatocytes. The remarkably broad range of specific biological processes that are shown here to be perturbed by the loss of N-terminal arginylation will make possible the dissection of regulatory circuits that involve Ate1 and either its known substrates, such as Rgs4, Rgs5 and Rgs16, or those currently unknown.

## Introduction

**Figure 1:**
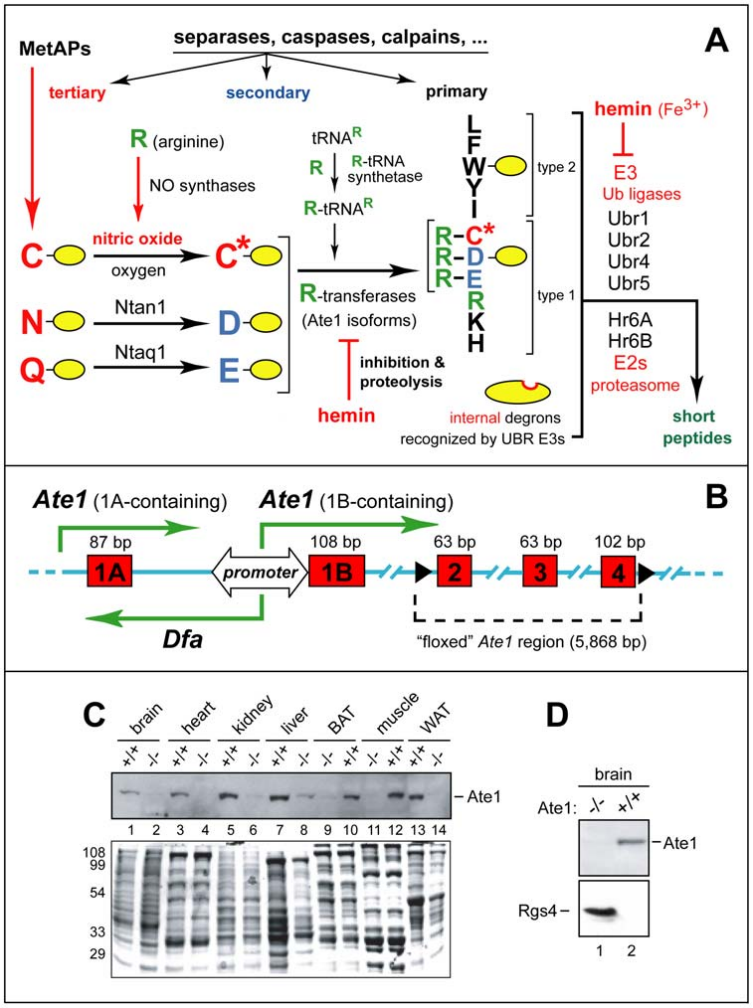
Postnatal ablation of the mouse Ate1 R-transferase, a component of the N-end rule pathway. (A) The mammalian N-end rule pathway. N-terminal residues are indicated by single-letter abbreviations for amino acids. Yellow ovals denote the rest of a protein substrate. “Primary”, “secondary” and “tertiary” denote mechanistically distinct subsets of destabilizing N-terminal residues (see Introduction). C* denotes oxidized Cys, either Cys-sulfinate or Cys-sulfonate. MetAPs, Met-aminopeptidases. (B) Bidirectional promoter between the mouse *Ate1* exons 1A and 1B [14]. Green arrows indicate transcriptional units, including a previously uncharacterized gene, termed *Dfa* (“divergent of Ate1), that is transcribed from the bidirectional promoter. (C) Immunoblotting-based comparisons of Ate1 levels in the indicated mouse tissues from *Ate1^+/+^* and *Ate1^flox/−^*;*CaggCreER* mice 76 days after the tamoxifen (TM)-induced, Cre-mediated *Ate1^flox^*→*Ate1^−^* conversion that yielded Ate1-deficient mice. The band of 60-kDa Ate1, detected by antibody to mouse Ate1, is indicated on the right. Total (Ponceau-stained) protein patterns are shown below, with positions of molecular-mass markers on the left. (D) IB assays for the levels of Ate1 and Rgs4 (25 kDa) in brain extracts from *Ate1^+/+^* and Ate1-deficient mice (*Ate1^flox/−^*;*CaggCreER* mice 30 days after TM treatment).

N-terminal arginylation of intracellular proteins by Arg-tRNA-protein transferase (R-transferase) is a part of the N-end rule pathway of protein degradation (Fig. 1A). In eukaryotes, this pathway is a part of the ubiquitin (Ub)-proteasome system. The N-end rule relates the *in vivo* half-life of a protein to the identity of its N-terminal residue (reviewed in [1], [2], [3], [4]). Degradation signals (degrons) that can be targeted by the N-end rule pathway are of two distinct kinds: N-terminal degrons, called N-degrons, and internal (non-N-terminal) degrons [1], [5]. The main determinant of an N-degron is a destabilizing N-terminal residue of a substrate protein (Fig. 1A). The other determinants of N-degron are a substrate's internal Lys residue (the site of formation of a poly-Ub chain) and a nearby unstructured region [6], [7]. An N-degron is produced from a precursor, called a pre-N-degron, through a protease-mediated cleavage of a substrate that exposes a destabilizing N-terminal residue.

The N-end rule has a hierarchic structure (Fig. 1A). N-terminal Asn and Gln are tertiary destabilizing residues in that they function through their enzymatic deamidation, to yield the secondary destabilizing N-terminal residues Asp and Glu [8]. Destabilizing activity of N-terminal Asp and Glu requires their conjugation to Arg, one of the primary destabilizing residues, by the *Ate1*-encoded R-transferase [9], [10], [11], [12]. In eukaryotes that produce nitric oxide (NO), R-transferase arginylates not only N-terminal Asp and Glu but also Cys, after its conversion to Cys-sulfinate or Cys-sulfonate, in reactions that require NO and oxygen (Fig. 1A) [11], [13]. Alternative splicing of the mammalian *Ate1* pre-mRNA produces isoforms of R-transferase, a metabolically unstable protein whose enzymatic activity and the in vivo half-life are down-regulated by heme [10], [12], [14]. E3 Ub ligases of the N-end rule pathway are called N-recognins. An N-recognin is an E3 that can recognize (target for polyubiquitylation) at least a subset of N-degrons (Fig. 1A) [1], [4]. Some of substrate-binding sites of an N-recognin target N-degrons, while other sites of the same N-recognin are specific for structurally unrelated internal (non-N-terminal) degrons [15], [16]. At least four N-recognins, Ubr1, Ubr2, Ubr4 and Ubr5, mediate the mammalian N-end rule pathway (Fig. 1A) [4], [17].

The functions of the N-end rule pathway in eukaryotes include selective degradation of misfolded proteins; the sensing of heme, oxygen, nitric oxide (NO), and short peptides; the regulation of DNA repair and peptide import; the signaling by transmembrane receptors, through the NO/O_2_-controlled degradation of G-protein regulators Rgs4, Rgs5 and Rgs16; the fidelity of chromosome segregation; regulation of apoptosis, meiosis, spermatogenesis, neurogenesis, and cardiovascular development; the functioning of specific organs, in particular the brain and the pancreas; and regulation of leaf senescence, seed germination, and other processes in plants ([2], [12], [16], [18], [19], [20], and refs. therein). A partial N-terminal arginylation of the apparently long-lived mammalian α-actin [21] suggests that arginylation of some proteins may not alter their *in vivo* half-lives.

Although there are many putative intracellular substrates of the Ate1 R-transferase, for example, among C-terminal fragments of proteins that are cleaved in vivo by proteases such as MetAPs, caspases, calpains or secretases, the set of definitively identified Ate1 substrates is still small. It includes the Drosophila antiapoptotic Ub ligase DIAP1 [22]; the mammalian G-protein regulators Rgs4, Rgs5 and Rgs16 [11], [13]; and the separase-produced fragment of the mammalian Rad21/Scc1 cohesin subunit that bears N-terminal Glu, a secondary destabilizing residue (Fig. 1A) (J. Zhou, D. Pati and A.V., unpublished data) [23]. Heterozygous *Ate1^+/−^* mice appear indistinguishable from their wild-type counterparts, whereas *Ate1^−/−^* mice die around embryonic day 15 (E15) with abnormalities that include cardiovascular defects [10].

To bypass the embryonic lethality of nonconditional *Ate1^−/−^* mice, we employed the Cre-lox technique [24]. As shown below, a systemic postnatal deletion of the sole active *Ate1^flox^* allele in juvenile *Ate1^flox/−^* mice causes a rapid decrease of body weight and results in early death of ∼15% of Ate1-deficient mice, with surviving mice attaining only ∼70% of normal weight. This failure to thrive occurs despite higher than normal food intake by Ate1-deficient mice. These mice contain little or no visceral fat, exhibit an increased metabolic rate, a decreased fasting blood glucose level, and an increased intestinal import and retention of amino acids and/or peptides. Ate1-deficient mice are also resistant to diet-induced obesity and exhibit ectopic induction of the Ucp1 uncoupling protein in white adipose tissue (WAT). In addition, Ate1-deficient mice have enlarged brains, an enhanced startle response, and are strikingly hyperkinetic. They often suffer from kyphosis, i.e., an excessive curvature of the upper back, and from frequent seizures as well. Ate1-deficient males are also infertile, owing to defects in meiotic *Ate1^−/−^* spermatocytes. The remarkably broad range of specific biological processes that are shown here to be perturbed by the loss of N-terminal arginylation will facilitate the dissection of regulatory circuits that involve Ate1 and either its known substrates, such as Rgs4, Rgs5 and Rgs16 [11], [13], or those currently unknown.

## Results

### *Ate1^flox/−^* Mouse Strains and Production of *Ate1^−/−^* Mice

**Figure 2:**
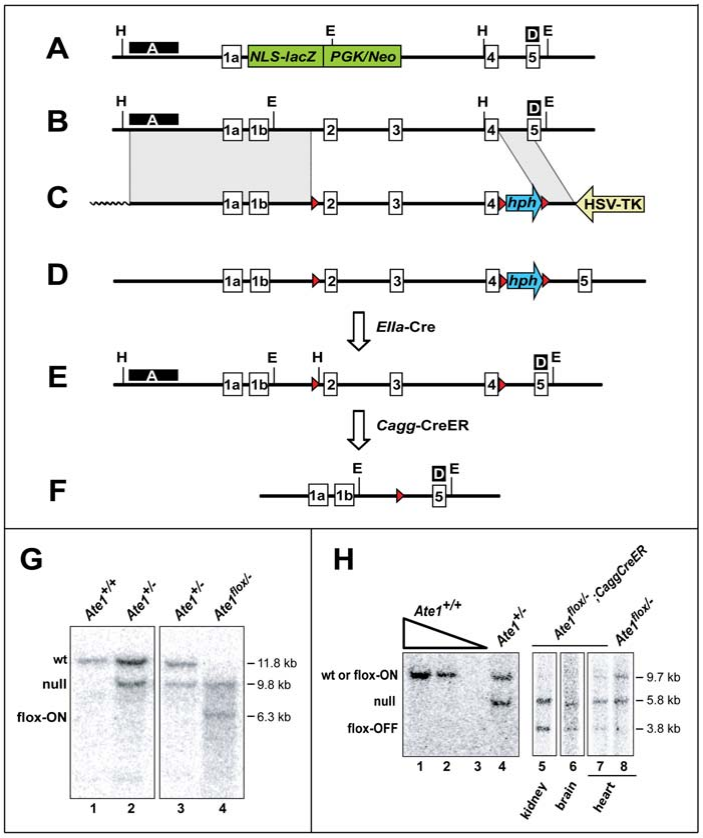
Genomic configurations at the *Ate1* locus of *Cre-lox*-based mouse strains constructed in the present work. (A) The 5′ end of the previously produced unconditional *Ate1^−^* allele [10], in which the *Ate1* exons 1b through 3 were replaced by a cassette encoding a promoter-lacking, NLS-containing LacZ (NLS-βgal) (it was expressed from the endogenous P*_Ate1_* promoter) and the Neo selection marker expressed from the phosphoglycerate kinase P*_PGK_* promoter (green rectangles). (B) A diagram of the 5′ end of wild-type (wt) mouse *Ate1*, indicating approximate locations of exons 1a through 5. (C) The ∼22.5 kb targeting construct containing a ∼6 kb long-arm region of *Ate1* homology (shown as a shaded rectangle on the left); a single loxP site (red triangle) upstream of *Ate1* exon 2, a “floxed”-hygromycin-resistance (*hph*) cassette, expressed from the P*_PGK_* promoter (blue arrow between two red triangles) downstream of *Ate1* exon 4; a ∼2 kb short-arm region of homology (an inclined shaded rectangle), and the HSV thymidine kinase (tk) negative-selection cassette expressed from the P*_HSV_* promoter (yellow arrow). Wavy line indicates an abutting sequence of the pBR322 plasmid DNA. (D) The tri-lox *Ate1* allele obtained after a correctly targeted double crossover event. (E) In the notations here and elsewhere in the paper, “flox-on” indicates a configuration depicted in this panel (the functionally active *Ate1^flox^* allele), whereas “flox-off” indicates a configuration depicted in panel F (the null *Ate1^−^* allele). The functionally active, “flox-on” (*Ate1^flox^*) allele, obtained by the removal of the *hph* cassette, using the *in vivo* expression of Cre-recombinase driven by the P*_EIIA_* promoter, which is active only in pre-implantation blastocysts. (F) The null “flox-off” (*Ate1^−^*) allele obtained by the inducible expression of CreER recombinase from the P*_Cagg_* promoter and posttranslationally induced by tamoxifen (TM) treatment (see the main text and Materials and Methods). H, approximate locations of HindIII sites used in Southern analyses with DNA probe **A** (see panel G); E, approximate locations of EcoRI sites used in Southern analyses with DNA probe **D** (see panel H); black boxes marked “A” and “D” indicate the regions specific for DNA probes **A** and **D**, respectively. (G) Southern hybridization analysis using DNA probe **A** and HindIII-digested genomic DNA. The wt *Ate1* allele (panel B) yields the 11.8 kb HindIII fragment. The previously constructed [10] unconditionally null *Ate1^−^* allele (panel A), denoted as “null” on this panel, yields the 9.8 kb HindIII fragment. The functionally active flox-on (*Ate1^flox^*) allele (panel E) yields the 6.3 kb HindIII fragment. Lane 1, *Ate1^+/+^*; lane 2, *Ate1^+/−^*; lane 3, *Ate1^+/−^*; lane 4, *Ate1^flox/−^*. (H) Southern hybridization analysis using DNA probe **D** (external to targeting vector) and EcoRI-digested genomic DNA. The previously constructed [10] unconditionally null *Ate1^−^* allele (denoted as “null”) yields the 5.8 kb fragment. Both the wild-type *Ate1* allele and the flox-on (*Ate1^flox^*) allele yield the 9.7 kB fragment, whereas the null flox-off (*Ate1^−^*) allele yields the characteristic 3.8 kb fragment. The use of DNA probe **D** and EcoRI-digested DNA from specific tissues of tamoxifen (TM)-treated *Ate1^flox/−^*;*CaggCreER* mice allowed approximate estimates of the levels of Cre-mediated recombination that produced the flox-off (*Ate1^−^*) allele. For example, whereas no flox-on (*Ate1^flox^*) allele could be detected in the kidney and brain of *Ate1^flox/−^*;*CaggCreER* mice after TM treatment (lanes 5, 6), approximately equal amounts of flox-on (*Ate1^flox^*) and flox-off (*Ate1^−^*) alleles were present in the heart of TM-treated *Ate1^flox/−^*; *CaggCreER* mice. Lanes 1–3, 1,000, 250, and 25 ng of EcoRI-digested wt mouse genomic DNA (from a tail biopsy), respectively. Lane 4, EcoRI-digested genomic DNA from the tail of a previously constructed [10] *Ate1^+/−^* mouse. Lanes 5–7, EcoRI-digested genomic DNA from the indicated tissues of TM-treated *Ate1^flox/−^*;*CaggCreER* mice. Lane 8, same as lane 7, but from a TM-treated *Ate1^flox/−^* mouse (lacking the *CaggCreER* transgene).

Standard methods were employed to produce, initially, *ATE^flox/+^* mouse strains in which a specific segment of *Ate1* was “floxed”, i.e., flanked by 34-bp *loxP* repeats (Fig. 2C–E). The targeting vector contained ∼14 kb of *Ate1*, including the exon 1A-exon 4 segment that encodes an essential part of R-transferase [9] (Fig. 2A). Our previous work has shown that the *Ate1* promoter (P*_Ate1_*) is bidirectional, expressing both *Ate1* and an oppositely oriented gene termed *Dfa* (divergent from *Ate1*), which overlaps with exon 1A of *Ate1* (Fig. 1B) ([14]; C.S.B. and A.V., unpublished data). To minimize the possibility of perturbing the expression of *Dfa*, the “floxed” region of *Ate1* encompassed exons 2–4, away from exon 1A (Fig. 1B). Our aim was to produce *ATE^flox/−^* mouse strains that were “poised” to lose their remaining active *ATE^flox^* allele through the expression of Cre recombinase. To do so, heterozygous matings were carried out among the above *ATE^flox/+^* mice, the previously constructed *ATE^+/−^* mice [10], and a mouse strain that contained the *CaggCreER* gene, expressed from the ubiquitously active chimeric *Cagg* promoter [25]. *CaggCreER* encoded CreER, a fusion between Cre and a derivative of the mouse estrogen receptor ligand binding domain. CreER was functionally inactive (sequestered in the cytosol) but could be activated by intraperitoneal (IP) injections of tamoxifen (TM) [25]. Depending on configurations of their *Ate1* alleles, the resulting mice, poised for the loss of *Ate1*, were termed *Ate1^flox/−^*;*CaggCreER* or *Ate1^flox/flox^*;*CaggCreER*.

**Figure 3:**
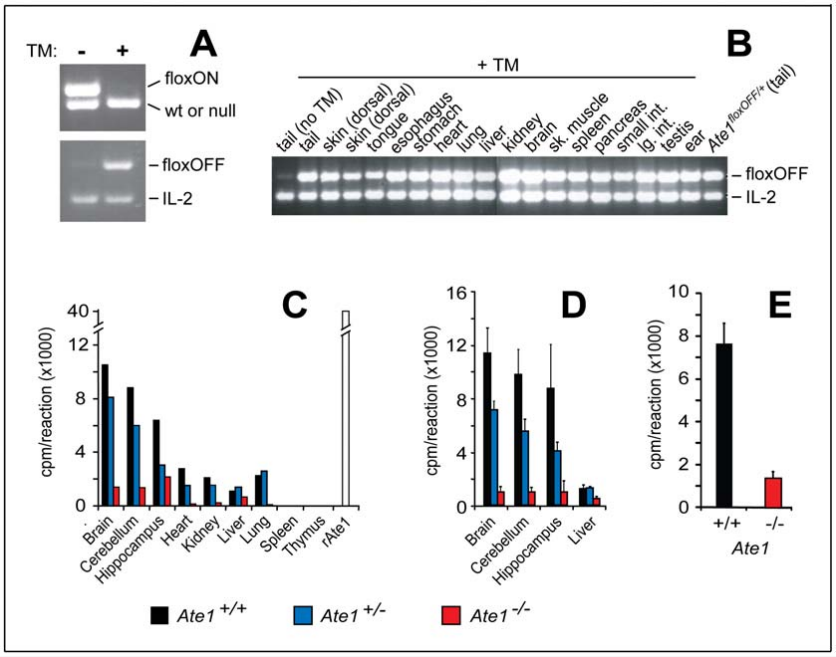
Cre-mediated conversion to *Ate1*-null genotype in different mouse tissues. (A) PCR-based genotyping of tail DNA to detect the Cre-mediated *Ate1^flox^*→*Ate1^−^* conversion of the functionally active flox-on (*Ate1^flox^*) allele to the null *Ate1^−^* allele in a 27-day old *Ate1^flox/−^*;*CaggCreER* mouse immediately after the fourth (daily) intraperitoneal (IP) injection of tamoxifen (TM+), or in the absence of TM treatment (TM-). Upper panel: the 512 bp DNA fragment characteristic of the flox-on (*Ate1^flox^*) allele and the 472 bp DNA fragment characteristic of either wild-type or the previously constructed [10] unconditionally null *Ate1^−^* allele, using primers CB156 and CB157 (Table 4). Lower panel: the 470 bp DNA fragment characteristic of the Cre-produced flox-off (*Ate1^−^*) allele, with primers CB110 and CB157 (Table 4); and the 324 bp DNA fragment (control), amplified from the *IL-2* gene using primers IMR42 and IMR43, in the same PCR reaction. (B) The Cre-mediated *Ate1^flox^*→*Ate1^−^* conversion, detected by PCR (as described in panel A) in genomic DNA isolated from the indicated tissues immediately after the fourth (daily) IP injection of tamoxifen in a 24-day old *Ate1^flox/−^*;*CaggCreER* mouse. (C) Relative in vitro arginylation activity (cpm/reaction) in extracts of the indicated tissues from a wild type mouse (*Ate1^+/+^*) (black bar), a heterozygous mouse (*Ate1^+/−^*) (blue bar), and an *Ate1^−/−^* mouse (the latter mouse was initially *Ate1^flox/−^*;*CaggCreER*) (red bar) from the same litter 76 days after TM treatment. A white bar on the right indicates the relative arginylation activity obtained with purified recombinant mouse Ate1 (denoted as “rAte1”) that had been expressed in S. cerevisiae. Shown here are “cpm/reaction” after subtracting “cpm/reaction” in the null-control (“buffer alone”) sample. The control incorporation was approximately equal to that observed in extracts from spleen and thymus. In other words, the assay configured as described in this panel and in Materials and Methods was not sensitive enough to robustly detect the arginylation activity in extracts from spleen and thymus. (D) Relative in vitro arginylation activity (cpm/reaction) in the whole brain, cerebellum, and hippocampus harvested from wild type mice (*Ate1^+/+^*; n = 3), heterozygous mice (*Ate1^+/−^*; n = 3), and *Ate1^−/−^* mice (specifically, *Ate1^flox/−^*;*CaggCreER* mice; n = 3) mice 40 days after TM treatment. Standard deviations are indicated. (E) Relative in vitro arginylation activity (cpm/reaction) in testis extracts from *Ate1^+/+^* mice (n = 3) and *Ate1^−/−^* mice (specifically, *Ate1^flox/−^*;*CaggCreER* mice; n = 3) ∼130 days after TM treatment. Standard deviations are indicated.

Using standard methods, we could demonstrate the presence of *Ate1^flox/−^*;*CaggCreER* mice, at expected (Mendelian) frequencies, in the progeny of above matings. These mice expressed TM-inducible CreER recombinase and contained a single copy of *Ate1^flox^*, the active *Ate1* allele (Figs. 2 and 3A). The functional intactness of *Ate1^flox^* was inferred from the fact that *Ate1^flox/−^*;*CaggCreER* mice survived embryogenesis (in contrast to *Ate1^−/−^* mice [10]) and were phenotypically similar (in the absence of TM treatment) to *Ate1^+/−^* and *Ate1^+/+^* mice. To induce the *Ate1^flox^*→*Ate1^−^* conversion, ∼1 month old *Ate1^flox/−^*;*CaggCreER* mice and their *Ate1^flox/+^*;*CaggCreER* (as well as *Ate1^+/−^*;*CaggCreER*) littermates, used as controls, were treated with TM (see Materials and Methods for details, including the ages of TM-treated mice). Southern hybridization and PCR-based analyses of DNA from tissues of the resulting mice (sampled ∼1 month after TM treatment) confirmed the TM-induced, Cre-mediated excision of the *Ate1^flox^* allele in *Ate1^flox/−^*;*CaggCreER* mice. The frequency of *Ate1^flox^*→*Ate1^−^* conversion was nearly 100% in the brain and kidney of these mice, but significantly lower in several other tissues (Figs. 2G, H and 3B).

We also used an affinity-purified antibody to mouse Ate1 [11] to carry out immunoblotting (IB) with extracts from brain, heart, kidney, liver, muscle, brown adipose tissue (BAT) and white adipose tissue (WAT) that were harvested up to 8 months after TM treatment of *Ate1^flox/−^*;*CaggCreER* mice, versus identically TM-treated control littermates. No Ate1 could be detected by IB in several tissues of TM-treated *Ate1^flox/−^*;*CaggCreER* mice, in contrast to readily detectable Ate1 in TM-treated control mice (Fig. 1C). The only significant exception was liver (Fig. 1C, lanes 7, 8; *cf*. lanes 5, 6 or lanes 11–14; see also below). One effect of Ate1 depletion in the mouse brain was a striking increase of Rgs4, a physiological Ate1 substrate (see Introduction) that down-regulates specific G proteins by acting as a GTPase-activating protein (GAP) (Fig. 1D). Whereas no Rgs4 could be detected in the Ate1-containing brain (owing to degradation of Rgs4 by the N-end rule pathway [11]), an intense band of Rgs4 was present in the Ate1-deficient brain, illustrating high penetrance of *Ate1* deletion in the brain (Fig. 1D).

**Figure 4:**
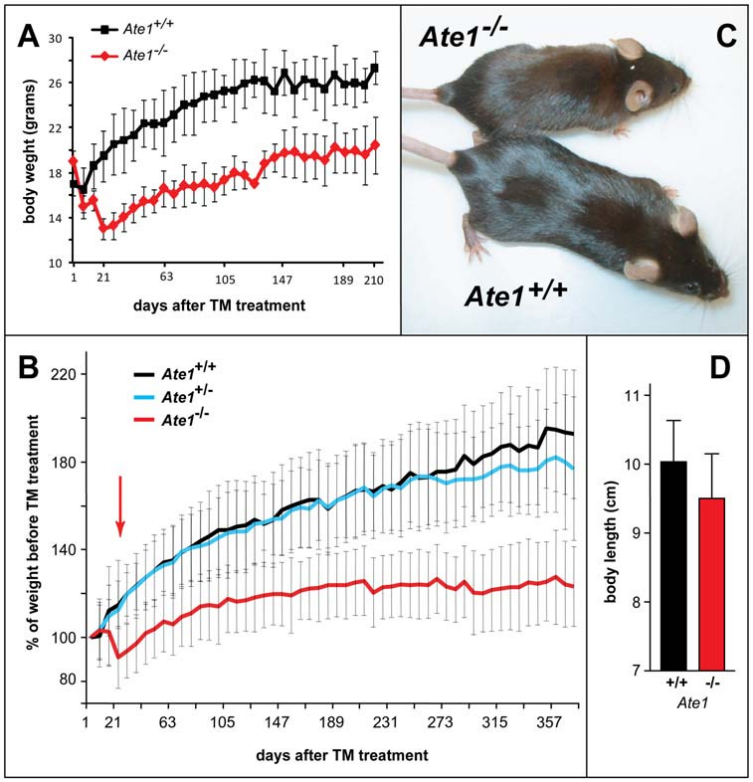
Growth rate consequences of postnatal ablation of Ate1. (A) Weights of Ate1-containing (n = 4; black curve) and Ate1-deficient (n = 2; red curve) mice from the same litter as a function of time after tamoxifen (TM) treatment. Weights were measured at weekly intervals. Vertical bars indicate the ranges of measured weights. (B) Averaged growth curves for the indicated numbers of mice after TM treatment, plotted as a percentage of their weight immediately before TM treatment. Red, black and blue curves: *Ate1^−/−^* (n = 87), *Ate1^+/+^* (n = 55), and *Ate1^+/−^* (n = 66) mice. Red arrow indicates the time (∼21 days) after TM treatment by which ∼15% of Ate1-deficient mice have died while the rest of them began to gain weight. Note a slightly but clearly decreased weight of heterozygous (*Ate1^+/−^*) mice (blue curve), in comparison to *Ate1^+/+^* mice (black curve) ∼1 year after TM treatment. Error bars indicate standard deviations (SD). (C) Typical appearance of *Ate1^−/−^* versus wt mice (a smaller, leaner *Ate1^−/−^* mouse) ∼1 year after TM-mediated ablation of *Ate1*. (D) Mean body lengths (± SD) (from tip-of-nose to base-of-tail) between pairs of *Ate1^−/−^* (red bar) and *Ate1^+/+^* (black bar) mice. This comparison was derived from the data in Fig. 5C. Statistical analysis was performed using an unpaired t-test (p<0.08).

We also performed in vitro arginylation assays with extracts from several tissues of *Ate1^flox/−^*;*CaggCreER* mice 21 days after TM treatment, versus extracts from identically treated *Ate1* ^+/+^ or *ATE1* ^+/−^ mice. The TM-induced decrease of arginylation activity in specific organs of *Ate1^−/−^*;*CaggCreER* mice ranged from ∼90% in the brain and kidney to ∼60% in the liver (Fig. 3C–E). Although heterozygous *Ate1* ^+/−^ mice were phenotypically similar to their wild-type (*Ate1* ^+/+^) counterparts, we found that *Ate1* ^+/−^ mice grew slightly but consistently slower than *Ate1* ^+/+^ mice, and reached a lower average weight (Fig. 4B). In agreement with this mild but detectable haploinsufficiency of *Ate1*, the arginylation activity in extracts from, e.g., brains or hearts of *Ate1* ^+/−^ mice was significantly below its wild-type (*Ate1* ^+/+^) levels (Fig. 3C), implying the absence of a compensatory (e.g., autoregulated) increase of *Ate1* expression upon a decrease of *Ate1* gene dosage.

### Retarded Growth, Kyphosis, and a Transient Increase in Lethality of Ate1-Deficient Mice

**Figure 5:**
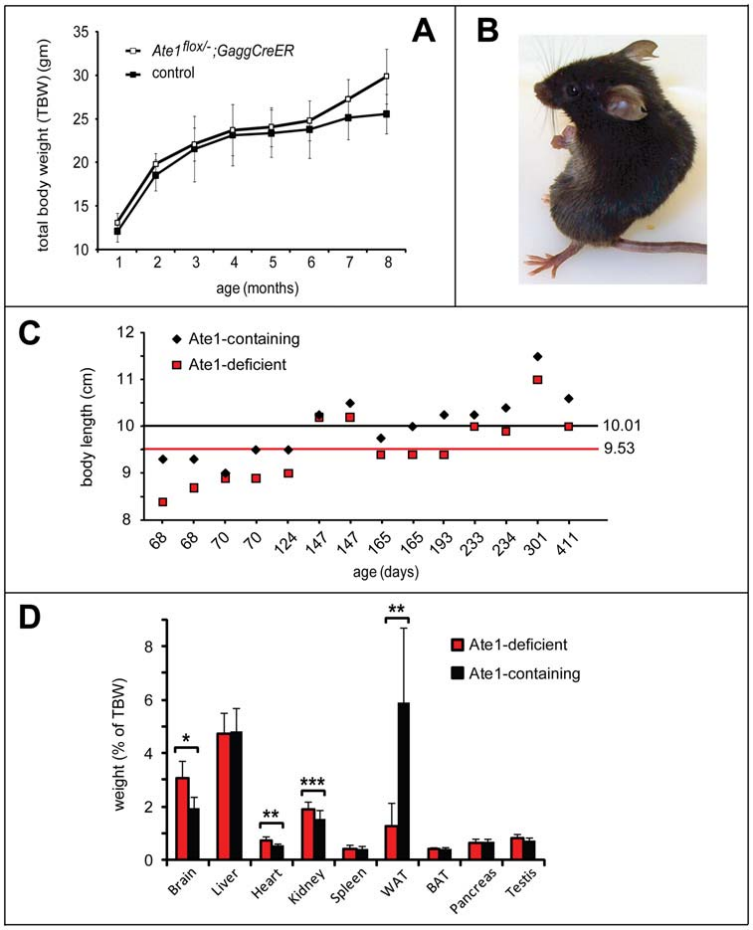
Comparison of organ sizes and other parameters of *Ate1^−/−^* versus *Ate1^+/+^* mice. (A) Averaged growth curves (total body weight (TBW)) for *Ate1^flox/−^*;*CaggCreER* mice versus control mice in the absence of TM treatments. A cohort of “control” mice contained *Ate1^flox/+^* mice (n = 2); *Ate1^flox/−^* mice (n = 2); *Ate1^+/+^* mice (n = 1) and *Ate1^+/+^*;*CaggCreER* mice (n = 1) from 1 month of age through 8 months. None of the mice were treated with TM. Vertical bars indicate standard deviations. (B) Typical “kyphoid” posture of an Ate1-deficient mouse (see also the main text). (C) A plot of body lengths (in cm from tip-of-nose to base-of tail) in individual sets of Ate1-containing (black diamonds) and Ate1-deficient (red boxes) siblings at the indicated ages. Each pair of symbols, at a given age, represents a single pair of siblings. The black horizontal line indicates the averaged body length of all Ate1-containing mice (n = 14). The red horizontal line indicates the averaged body length of all Ate1-deficient mice (n = 14). (D) Comparison of tissue weights (as a percentage of total body weight (TBW)). Numbers in parentheses indicate the numbers of mice sampled and averaged for each tissue (Ate1-containing and Ate1-deficient). Brain (n = 43), liver (n = 28), heart (n = 17), kidney (n = 17), spleen (n = 16), white adipose tissue (WAT; n = 10), brown adipose tissue (BAT; n = 10), pancreas (n = 6), and testis (n = 8) from Ate1-containing (black bars) and Ate1-deficient mice (red bars). *  =  p<8×10^−15^; **  =  p<5×10^−5^; and ***  =  p<0.003. Statistical analysis was performed using an unpaired t-test. Standard deviations are indicated.

TM treatment produced abnormal phenotypes within 1 week in *Ate1^flox/−^*;*CaggCreER* mice, in comparison to identically TM-treated controls. Specifically, ∼1 month old and previously growing *Ate1^flox/−^*;*CaggCreER* mice failed to thrive (in comparison to control mice) after their TM-induced conversion to *Ate1^−/−^*;*CaggCreER* mice (Figs. 4A–C and 5A). During the first ∼3 weeks after becoming *Ate1^−/−^*, these mice experienced a rapid loss of weight and decreased growth (Fig. 4A, B), despite no decrease in their consumption of food (see below). The average body length (measured from tip-of-nose to base-of-tail) of *Ate1^−/−^*;*CaggCreER* mice was 5% smaller (p<0.08) than that of their Ate1-containing, identically TM-treated counterparts (*Ate1^+/+^*;*CaggCreER*, *Ate1^+/−^*;*CaggCreER*, or *Ate1^flox/+^*;*CaggCreER* mice) (Figs. 4D and 5C).

In the entire cohort of TM-treated post-natal *Ate1^flox/−^*;*CaggCreER* mice, 15% of them (18 of 119 mice) died over 42 days after TM treatment. Crucially, none of identically TM-treated control mice (*Ate1^+/+^*;*CaggCreER*, *Ate1^+/−^*;*CaggCreER*, or *Ate1^flox/+^*;*CaggCreER*) died in the same time interval. The frequency of *Ate1^−/−^*;*CaggCreER* mice succumbing upon the acquisition of *Ate1^−/−^* genotype was age-dependent. Specifically, 46% of *Ate1^−/−^*;*CaggCreER* mice younger than 30 days at the beginning of TM treatment died within 42 days after TM treatment. In contrast, only 12% of *Ate1^−/−^*;*CaggCreER* mice died if they were older than 30 days (by up to 56 days) at the beginning of TM treatment. Those among *Ate1^−/−^*;*CaggCreER* mice that survived for at least 42 days after TM treatment eventually resumed growth, but the rate of growth and their maximum weight were significantly below those parameters for identically TM-treated control mice (Fig. 4A–C).

**Figure 6:**
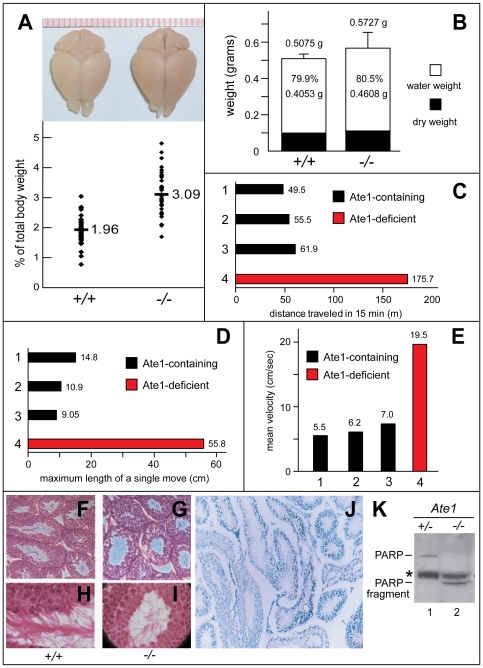
Brain, behavioral, and testis abnormalities of Ate1-deficient mice. (A) Enlarged brains of Ate1-deficient mice. Upper panel: comparison of representative brains harvested from an *Ate1^+/+^* and an *Ate1^−/−^* mouse, respectively, 134 days after tamoxifen (TM) treatment. Lower panel: brain weights expressed as percentages of total body weights in *Ate1^+/+^* (n = 41) and *Ate1^−/−^* (n = 40) mice. Horizontal bars and numbers indicate mean values. (B) Wet (0.4053 g versus 0.4608 g) and dry (0.1022 g versus 0.1119 g) weight components of the total mean brain weights (±SD) in *Ate1^+/+^* and *Ate1^−/−^* mice. (C) Total distance traveled (in meters), over 15 min, in an open field test among mice of different genotypes belonging to the same litter, 44 days after TM-treatment. Bar 1, *Ate1^flox/+^*;*CaggCreER* mouse. Bar 2, *Ate1^+/+^*;*CaggCreER* mouse. Bar 3, *Ate1^+/+^* mouse. Bar 4, *Ate1^flox/−^*;*CaggCreER* mouse that was converted to *Ate1^−/−^* by TM treatment. Blue and red bars denote Ate1-containing and Ate1-deficient mice, respectively. (D) Same as in C but maximum lengths of single movements (in centimeters). (E) Same as in C but mean velocities (in cm/second) over 15 min. (F) Paraffin sections (4 µm) of testis showing cross-sections of seminiferous tubules in *Ate1^+/+^* testis stained with hematoxylin and eosin (150× magnification). (G) Same as in F but *Ate1^−/−^* testis. Note that sperm tails in the lumens of *Ate1^−/−^* tubules are sparse in comparison to those in *Ate1^+/+^* testis. (H) Same as in F but at 600× magnification. (I) Same as in G but at 600× magnification. (J) XGal staining for βgal activity in a 10-µm section of *Ate1^+/−^* testis in which one copy of *Ate1* was replaced by an ORF encoding NLS-β-galactosidase and expressed from the P*_Ate1_* promoter (100× magnification). (K) Immunoblotting analysis, using antibody to poly (ADP-ribose) polymerase (PARP), of testis extracts from an Ate1-containing (*Ate1^flox/−^* (+/−)) and an Ate1-deficient (*Ate1^flox/−^*;*CaggCreER* (−/−)) mouse 16 days after TM treatment. Note the loss of the full-length length 116 kDa PARP and the presence of the 85 kDa PARP fragment (lane2). An asterisk denotes a protein crossreacting with anti-PARP antibody.

**Figure 7:**
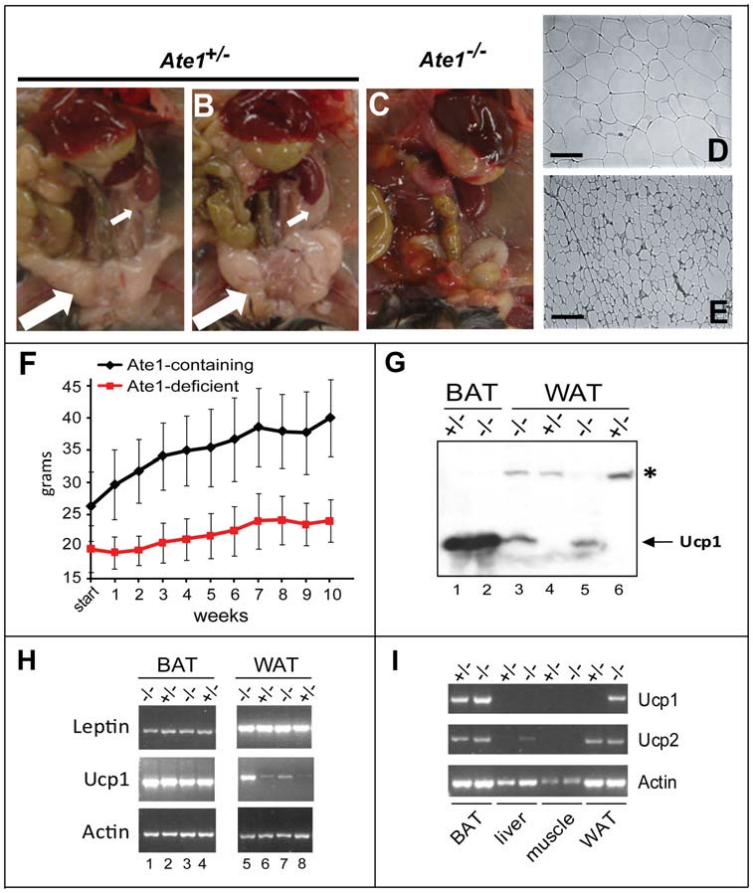
Loss of white adipose tissue (WAT), resistance to high fat diet-induced obesity, and ectopic Ucp1 in WAT of Ate1-deficient mice. (A–C) Visceral fat content of Ate1-containing mice. Shown here are representative examples of Ate1-containing (*Ate1^flox/+^*;*CaggCreER*) (A) and *Ate1^flox/−^* (B)) and Ate1-deficient (*Ate1^flox/−^*;*CaggCreER* (C)) mice 37 days after TM-treatment. Note the loss of both visceral fat (large white arrow in A and B) and fat surrounding the kidney (small white arrows in A and B) in an Ate1-deficient mouse (C). (D) Hematoxylin/eosin staining of a 10-µm section of white adipose tissue (WAT) harvested from an Ate1-containing mouse (TM-treated *Ate1^flox/+^*;*CaggCreER*). The bar denotes 100 µm. (E) Same as in D except that WAT was from an Ate1-deficient mouse (TM-treated *Ate1^flox/−^*;*CaggCreER*). (F) Average weights of TM-treated Ate1-containing (n = 12; black curve) and Ate1-deficient (n = 11; red curve) mice as a function of time after the beginning of *ad libitum* high-fat diet. Weights were measured at weekly intervals for 10 weeks. Error bars indicate ±SD. (G) Comparisons, by immunoblotting, of Ucp1 protein levels in extracts from brown adipose tissue (BAT) (lanes 1 and 2) and WAT (lanes 3 through 6) from *Ate1^+/−^* and *Ate1^−/−^* mice 46 days (lanes 1 and 2) or ∼1 year (lanes 3–6) after TM treatment. Specific genotypes were as follows (genotypes after TM treatment are indicated in parentheses here, and also on top of the gel): lane 1, *Ate1^flox/−^* (+/−); lane 2, *Ate1^flox/−^*;*CaggCreER* (−/−); lane 3, *Ate1^flox/−^*;*CaggCreER* (−/−); lane 4, *Ate1^flox/−^* (+/−); lane 5, *Ate1^flox/−^*;*CaggCreER* (−/−); lane 6, *Ate1^flox/+^*;*CaggCreER* (+/−). Note abnormally high expression of Ucp1 in WAT of Ate1-deficient mice (lanes 3 and 5). An asterisk denotes a protein in WAT that cross-reacts with anti-Ucp1 antibody. (H) RT-PCR analyses of leptin and *Ucp1* mRNA levels in BAT (lanes 1–4) and WAT (lanes 5–8) of Ate1-containing (denoted as “+/−”; lanes 2, 4, 6, and 8) and Ate1-deficient (denoted as “−/−”; lanes 1, 3, 5, and 7) mice ∼1 year after TM treatment. Specific genotypes: lanes 1 and 5, *Ate1^flox/flox^*;*CaggCreER* (−/−); lanes 2 and 6, *Ate1^flox/+^*;*CaggCreER* (+/−); lanes 3 and 5, *Ate1^flox/flox^*;*CaggCreER* (−/−); lanes 4 and 8, *Ate1^flox/−^* (+/−). (I) RT-PCR analyses of *Ucp1* and *Ucp2* mRNA levels in BAT, liver, muscle, and WAT of an *Ate1^flox/+^* mouse (denoted as “+/−”) and an *Ate1^flox/−^*;*CaggCreER* mouse (denoted as “−/−”)∼1 year after TM treatment.

In addition to their retarded growth (despite a higher than normal food intake; see below), 53% of *Ate1^−/−^*;*CaggCreER* mice (95 of 180 mice) appeared “scruffy”, and 66% of them (109 of 180) had a kyphotic posture, i.e., an excessive curvature of the upper back (Fig. 5B). In contrast, only 3% of Ate1-containing mice (8 of 244) were scruffy, and only 2% (5 of 244) exhibited kyphosis. Among surviving *Ate1^−/−^*;*CaggCreER* mice, 10% (8 of 80) developed patches of red hair among their normally black hair, in contrast to identically TM-treated Ate1-containing mice (data not shown), suggesting a misregulation of melanocytes in Ate1-deficient mice. The liver, spleen, intrascapular brown adipose tissue (BAT), pancreas, and testis of *Ate1^−/−^*;*CaggCreER* mice appeared normal and were of appropriate sizes (if the smaller size of these mice (Fig. 4C) was taken into account), whereas the brains, hearts and kidneys of these Ate1-deficient mice were disproportionately large, in comparison to those of Ate1-containing siblings (Fig. 5D). Intact brains of Ate1-deficient mice appeared swollen, in comparison to brains harvested, in parallel, from identically treated Ate1-containing siblings (Fig. 6A). In addition, Ate1-deficient males were infertile, in agreement with defects in their testes (Fig. 6F–I). Yet another abnormality of Ate1-deficient mice was their strikingly lower content of the peritoneal white adipose tissue (WAT), on average only 16% of WAT in Ate1-containing mice (Figs. 5D and 7A–C). These phenotypes are discussed below.

### Spermatogenesis Defects and Infertility of Ate1-Deficient Male Mice

The marking of *Ate1^−^* allele with NLS-β-galactosidase (βgal) expressed from the P*_Ate1_* promoter revealed high levels of Ate1 expression in the neural tube and other specific, often sharply delineated, regions of *Ate1^+/−^* embryos [10]. An earlier study detected high levels of *Ate1* expression in spermatogonia (stem cells, located at the periphery of testis' seminiferous tubules), and possibly also in early meiotic spermatocytes of adult mice [26]. Male *Ate1^−/−^*;*CaggCreER* mice that were produced by TM treatment (Fig. 4C) were found to be infertile in matings with Ate1-containing females, in contrast to identically TM-treated Ate1-containing males (data not shown). XGal staining of testis sections of NLS-βgal-marked *Ate1* ^+/*−*^ mice in the present work (Fig. 6J) confirmed and extended the earlier evidence [26] for the pattern of *Ate1* expression in testis. Whereas the lumens of seminiferous tubules in Ate1-containing testis were filled with inward-pointing sperm tails, the lumens of tubules in Ate1-deficient testis contained few sperm cells, in a disorganized arrangement (Fig. 6F–I), in agreement with the observed infertility of Ate1-deficient males.

To address the timing of requirement for Ate1 during spermatogenesis, we mated wild-type females with *Ate1^flox/−^* males that contained (instead of the *CaggCreER* gene) the *PrpCreER* gene (line 28.8) [27] or the *PrmCre* gene [28]. *PrpCreER* expresses TM-inducible CreER from the *Prp* promoter, whose activity in testis is confined to spermatogonia and meiotic spermatocytes [27]. In contrast, *PrmCre* expresses the (unconditionally active) Cre recombinase from the protamine promoter, which is active at later stages of spermatogenesis, in (haploid) round and elongating spermatids [28]. Three breeding pairs for each of two kinds of *Ate1^flox/−^* males (*PrpCreER*-based and *PrmCre*-based) and wild-type females were set up. 33% fewer litters and 50% fewer pups were produced with *Ate1^flox/−^*;*PrpCreER* males, in comparison to *Ate1^flox/−^*;*PrmCre* males (Table 1). (This substantial difference is expected to be even larger in a setting where an expressed Cre does not require a second, TM-mediated step for activation, as is the case with TM-independent Cre expressed from the *Prm* promoter, but not with TM-inducible CreER, expressed from the *Prp* promoter.) Nearly equal numbers of the *Ate1^flox^* (active) and *Ate1^−^* (inactive) alleles were present in the heterozygous progeny of matings that involved *Ate1^flox/−^*;*PrmCre* males (13 versus 14 pups containing *Ate1^flox^* versus *Ate1^−^* alleles, respectively). In contrast and most revealingly, only one *Ate1^−^* (inactive) allele but 12 *Ate1^flox^* (active) alleles were present in the progeny of matings that involved *Ate1^flox/−^*;*PrpCreER* males (Table 1). These findings suggest that the *PrmCre*-mediated inactivation of the *Ate1^flox^* allele, which occurs at a post-meiotic stage of spermatogenesis [28], takes place at a time when Ate1 is no longer essential for production of viable sperm cells, thus accounting for high frequency of the *Ate1^−^* allele in the progeny of matings that involve *Ate1^flox/−^*;*PrmCre* males. In contrast, the *PrpCreER*-mediated inactivation of the *Ate1^flox^* allele, which takes place in meiotic spermatocytes [27], clearly discriminated against the transmission of the *Ate1^−^* allele, in comparison to the *Ate1^flox^* (active) allele, most likely because spermatocytes that became Ate1-deficient before they became haploid were sufficiently perturbed by the absence of arginylation to either undergo apoptosis or differentiate abnormally, yielding defective sperm cells.

**Table 1:** Genotypes of mice from matings of *Ate1^+/+^* females with *Ate1^flox/−^* males containing testis-specific *Cre* transgenes.

	Ate1^flox/−^;PrmCre ♂ x wild type ♀	Ate1^flox/−^;Prp28.8Cre ♂ x wild type ♀
# breeding pairs	3	3
# litters	6	4
Average litter size	7	5.25
total pups	42	21
# floxOFF	14	1
% floxOFF	33	4.7
# floxON	13	12
% floxON	31	57

Matings involving the Prp28.8 Cre strain occurred ∼1 month following TM treatment.

Previous work demonstrated a defective assembly of synaptonemal complexes and massive apoptosis of spermatocytes in *Ubr2^−/−^* mice [26]. The Ate1 R-transferase acts upstream of Ubr2 and other Ub ligases of the N-end rule pathway (Fig. 1A). Given a role of Ate1 in spermatogenesis demonstrated in the present study, it is possible that the currently unknown N-end rule substrate(s) whose degradation is in down-regulated in *Ubr2^−/−^* spermatocytes is an Ate1 substrate. To assess the extent of apoptosis in Ate1-deficient spermatocytes, we employed immunoblotting with antibody to poly(ADP-ribose)-polymerase (PARP), which is cleaved by caspases late in apoptosis. Anti-PARP antibody detected the (expected) 116 kDa full-length PARP in extracts from Ate1-containing mouse testis, but no 85-kDa PARP fragment, a marker of apoptosis (Fig. 6K, lane 1) [29]. In contrast, Ate1-deficient testis contained the 85-kDa fragment of PARP but virtually no full-length PARP, indicating extensive apoptosis in the absence of Ate1 (Fig. 6K, lane 2; *cf*. lane 1), in agreement with cytological and *Ate1^−/−^* male-infertility data (Fig. 6F–I). The 85-kDa PARP fragment is expected to bear N-terminal Gly [29], which is not a substrate of the Ate1 R-transferase (Fig. 1A). Thus the absence of the 85-kDa PARP fragment in Ate1-containing testis (Fig. 6K, lane 1) signifies the lack of production of this fragment by caspases, rather than its degradation by the arginylation branch of the N-end rule pathway. Proteins that require N-terminal arginylation for their degradation and that are likely to be relevant to meiotic functions of Ate1 include Rec8 [30], [31], a subunit of meiotic cohesin whose cleavage by separase is expected to produce an Ate1 substrate, similarly to the cleavage of Scc1/Rad21, the somatic counterpart of Rec8 (see Introduction).

### Hyperkinesia, Seizures, and Enlarged Brains of Ate1-Deficient Mice

**Figure 8:**
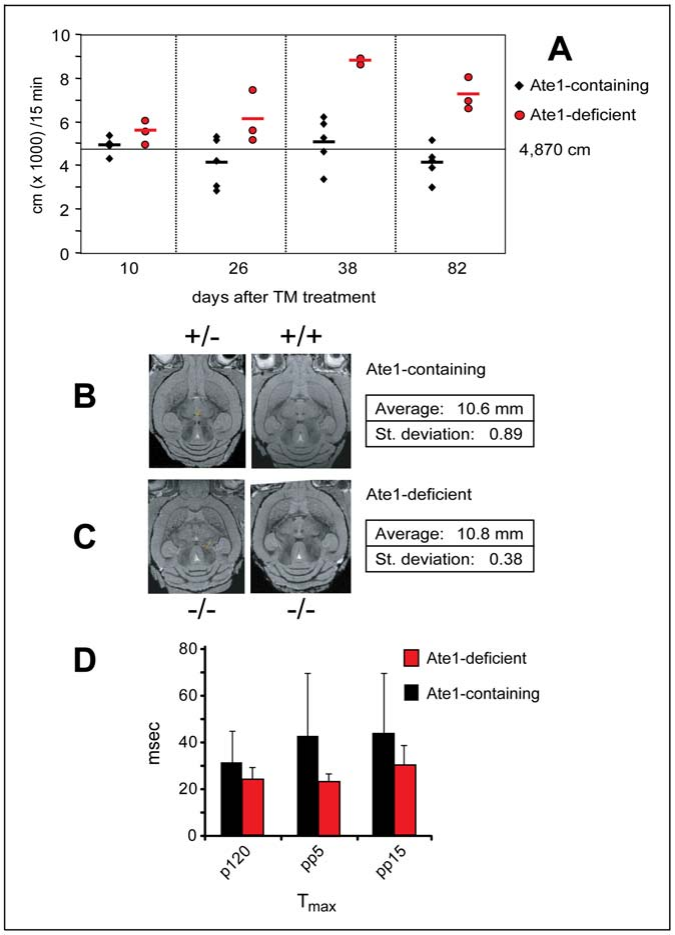
Brain abnormalities and behavioral phenotypes of Ate1-deficient mice. (A) Ate1-deficient mice become hyperactive as a function of time after TM-mediated ablation of *Ate1*. Total distance (in cm) traveled over 15 min in the open field test box (2500 cm^2^). This test was repeated every ∼2 weeks after the end of TM treatment. The data for Ate1-containing mice (n = 5; their genotypes were *Ate1^flox/+^*, *Ate1^flox/−^*, and *Ate1^flox/+^*;*CaggCreER*) and Ate1-deficient mice (n = 3; *Ate1^flox/−^*;*CaggCreER*) are indicated by black diamonds and red circles, respectively. The horizontal bars indicate mean values. The average total distance traveled over 15 min for all Ate1-containing mice (n = 37) was 4,870 cm. (B) Representative magnetic resonance images showing equivalent horizontal planes of Ate1-containing (*Ate1^flox/+^*;*CaggCreER* on the left, *Ate1^flox/+^* on the right) brains ∼3 months after TM treatment. The indicated average width of the skull (measured at the widest point from left to right in the same plane) of four Ate1-containing mice was 10.6 mm (±0.89 mm). (C) Same as in B except with brains from Ate1-deficient (*Ate1^flox/−^*;*CaggCreER*) mice ∼3 months after TM-treatment. The average width of the skull (measured as in B) of four Ate1-deficient mice was 10.8 mm (±0.38 mm). (D) Comparison of the response latency (T_max_; recorded in msec) between Ate1-containing (n = 3; black bars) and Ate1-deficient mice (n = 3; red bars) to a 40-msec pulse of 120 dB (p120; p<0.3), a 40-msec pulse of 120 dB preceded by a pre-pulse of 5 dB (pp5; p<0.09), or a 40-msec pulse of 120 dB preceded by a pre-pulse of 15 dB (pp15; p<0.01). Statistical analysis was performed using an unpaired t-test.

Most of *Ate1^−/−^*;*CaggCreER* mice (96 of 180) were strikingly hyperactive (hyperkinetic) (Figs. 6C–E and 8A). Intact brains harvested from Ate1-deficient mice appeared swollen, in comparison to brains harvested, in parallel, from Ate1-containing siblings (Fig. 6A). While the average brain weight, as a percentage of total body weight (TBW), of Ate1-containing mice was 1.96%, that of *Ate1^−/−^*;*CaggCreER* mice was 3.09% (Fig. 6A). In addition, there was a larger scatter of relative brain weights for Ate1-deficient mice, in comparison to identically TM-treated Ate1-containing controls. In particular, the brains of some Ate1-deficient mice reached 5% of TBW (Fig. 6A). Histological patterns of NLS-βgal [10] expressed from the P*_Ate1_* promoter in the brains of *Ate1^+/−^* mice (data not shown) were in agreement with in situ hybridization data in the Allen Brain Atlas (http://www.brain-map.org/), in that *Ate1* was expressed at varying but significant levels throughout the mouse brain, particularly in the hippocampus, dorsal thalamus, and cerebellum. No Ate1 protein could be detected in brain extracts of *Ate1^−/−^*;*CaggCreER* mice, in contrast to extracts from wild-type or *Ate1^+/−^* brains (Fig. 1C, D). The virtually null *Ate1* state of the brain in *Ate1^−/−^*;*CaggCreER* mice was also indicated by a strong accumulation of Rgs4, a physiological substrate of Ate1 (see Introduction) (Fig. 1D).

We carried out cell proliferation assays with *Ate1^−/−^*;*CaggCreER* mice (and controls), using 5-ethynyl–2′-deoxyuridine (EdU). In examinations of EdU-labeled brain sections, we paid particular attention to regions such as the hippocampus and the periventricular zone of the lateral ventricles, where neurogenesis is known to occur. However, no differences in EdU incorporation between Ate1-deficient and Ate1-containing brains were observed (data not shown), consistent with a brain edema (fluid accumulation) being a significant cause of brain enlargement in Ate1-deficient mice. We also determined the water content of freshly isolated brains, by subtracting their “dry weights” (after freeze-drying) from their total weights. The average water content and dry weight of control (Ate1-containing) brains was 79.9% and 20.1%, respectively, versus 80.5% and 19.5%, respectively (p<0.03), for Ate1-deficient brains (Fig. 6B). Thus cerebral edema at least contributes to the observed differences in brain weight between Ate1-deficient and Ate1-containing mice. It remains to be determined whether an edema (owing, e.g., to an osmotic imbalance or inflammation) suffices to account for consistently observed Ate1-dependent differences in brain weights (Fig. 6A, B).

There was also a 10-fold higher propensity for seizures among Ate1-deficient mice. For example, during routine cage changes and handling of mice, ∼3.1% of Ate1-deficient mice (38 of 1,232) versus ∼0.3% of identically TM-treated Ate1-containing mice had tonic-clonic seizures. The skulls of Ate1-deficient mice appeared to be thinner, “softer” than the sculls of Ate1-containing mice. Although MRI analyses did not reveal statistically significant abnormalities in the shape or size of skulls in Ate1-deficient mice (Fig. 8B, C), the MRI data did not preclude the possibility that bone structure may be perturbed in the absence of Ate1. These issues remain to be addressed.

The neurological/behavioral abnormalities of Ate1-deficient mice included an enhanced startle response, a marker for increased anxiety in rodents. Specifically, the latency between stimulus and response (T_max_) for Ate1-deficient mice was between 54% and 76% of the average latency for Ate1-containing controls, i.e., Ate1-deficient mice reacted significantly faster (Fig. 8D), thus exhibiting an enhanced startle response. The open field test is used to assess locomotor, exploratory and anxiety-like behavior in rodents. This test revealed a remarkably hyperkinetic behavior of Ate1-deficient mice (Figs. 6C–E and 8A), consistent with their enhanced startled response (Fig. 8D). The initial test involved a 15-min comparison of movements of Ate1-deficient mice versus Ate1-containing siblings of the same litter. An Ate1-deficient mouse traveled, during the test, a 3-fold greater distance than their (identically TM-treated) Ate1-containing counterpart (175.71 m versus 55.63 m, respectively) (Fig. 6C). The mean velocity of an *Ate1^−/−^*;*CaggCreER* mouse was 19.5 cm/sec, in comparison to 7.0 cm/sec for a wild-type (*Ate1* ^+/+^) mouse, 6.2 cm/sec for an *Ate1^+/+^*;*CaggCreER* mouse, and 5.5 cm/sec for an *Ate1^flox/+^*;*CaggCreER* mouse (Fig. 6E).

To assess generality of this striking phenotype, we repeated the open field test with three Ate1-deficient mice at 10, 26, 38, and 82 days after TM treatment, in parallel with TM-treated Ate1-containing (control) mice. At 10 days after TM treatment, i.e., soon after the acquisition of the *Ate1^−/−^* genotype, the differences between distances travelled by Ate1-deficient versus Ate1-containing mice were small (Fig. 8A). However, by 26 days after TM treatment, there was a statistically significant difference between Ate1-deficient and Ate1-containing mice in regard to their locomotor activity (Fig. 8A). By 82 days after TM treatment, the locomotor activity of Ate1-deficient mice, in conjunction with their elevated overall anxiety, increased so much that the device in which the open field tests were performed became nearly impractical, as Ate1-deficient mice (in contrast to Ate-containing ones) kept jumping out of the testing box.

### Depletion of White Adipose Tissue in Ate1-Deficient Mice, and Their Resistance to Diet-Induced Obesity

**Figure 9:**
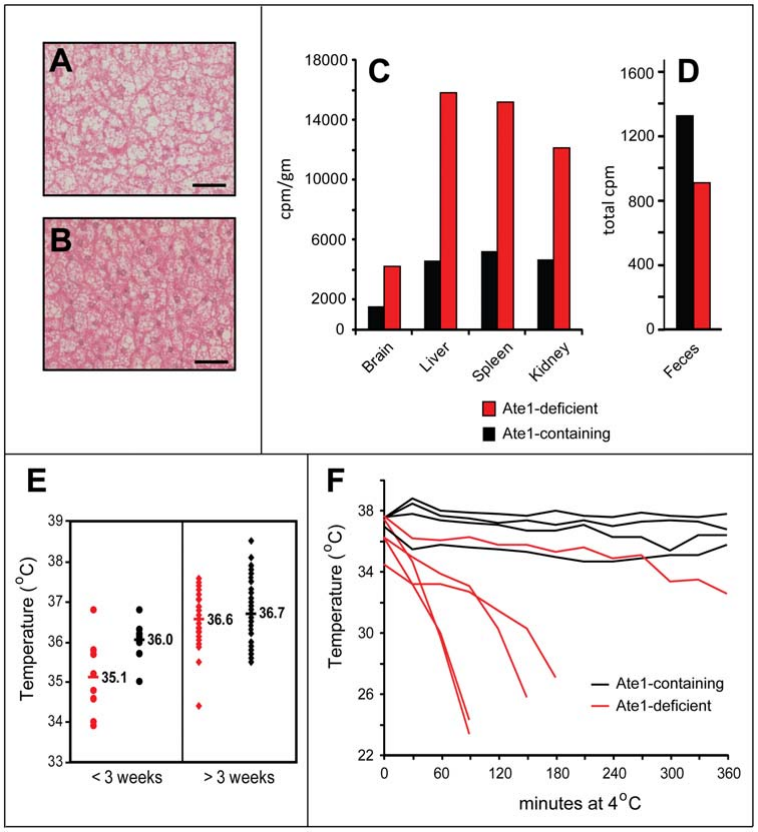
Body temperature, amino acid utilization and other properties of *Ate1^−/−^* versus *Ate1^+/+^* mice. (A) Hematoxylin/eosin staining (200× magnification) of a 10-µm section of brown adipose tissue (BAT) harvested from an Ate1-containing mouse (TM-treated *Ate1^flox/+^*;*CaggCreER*). The bar denotes 100 µm. (B) Same as in A except that BAT was from an Ate1-deficient mouse (TM-treated *Ate1^flox/−^*;*CaggCreER*). (C) Relative efficiencies of the import of ^14^C-amino acids and/or peptides from gastrointestinal tract in an Ate1-containing mouse (black bars; *Ate1^flox/+^*;*CaggCreER*) versus an Ate1-deficient mouse (red bars; *Ate1^flox/−^*;*CaggCreER*) 26 days after TM treatment. Shown here are representative comparisons of the retention of ^14^C (in cpm/gm) in the brains, livers, spleens, and kidneys 48 hr after gavage with a single bolus of ^14^C-labeled proteins (see Materials and Methods). (D) Total ^14^C (cpm) in the feces produced by mice in C within the first 48 hr after gavage with a bolus of ^14^C-labeled proteins. (E) Average core body temperatures of Ate1-containing (n = 8; black circles) versus Ate1-deficient (n = 11; red circles) mice during the first 3 weeks after TM treatment, in comparison to average core body temperatures of Ate1-containing (n = 54; black diamonds) versus Ate1-deficient (n = 36; red diamonds) mice beyond the first 3 weeks after TM treatment. (F) Core body temperature of individual Ate1-containing (black curves) and Ate1-deficient (red curves) mice, recorded at 30-min intervals after placing mice in a room at 4°C. Mice were removed from the cold room after 6 hr or when their core body temperature fell below 28°C.

To address the cause of a strikingly lower content of the peritoneal white adipose tissue (WAT) in Ate1-deficient mice, on average only 16% of WAT in Ate1-containing mice (Figs. 5D and 7A–C), we examined sections of intraperitoneal WAT. The average diameter of WAT adipocytes from Ate1-deficient mice was ∼30% of the average diameter of such cells in identically TM-treated Ate1-containing mice (25.5±7.4 µm versus 76.2±16.2 µm, respectively) (Fig. 7D, E). Thus, at least the bulk of WAT decrease in Ate1-deficient mice resulted from a decreased lipid content of individual adipocytes, rather from an extensive loss of adipocytes. Similar results were obtained with intrascapular brown adipose tissue (BAT) (Fig. 9A, B). The leanness of Ate1-deficient mice was particularly striking in view of their hyperphagy (see below).

We also asked whether the consistent difference in weight between Ate1-deficient and Ate1-containing mice on a standard ad libitum diet (Fig. 4A–C) could be reduced by an energy-rich, high-fat diet (HFD). At the end of the resulting 10-week test, the average weight of HFD-treated Ate1-containing mice was 152% of their starting weight (40.0 g versus 26.3 g). In contrast, the average weight of identically HFD-treated Ate1-deficient mice was only 122% (24.0 g versus 19.7 g) of their starting weight (Fig. 7F), indicating their relative resistance to diet-induced obesity. Yet another phenotype of Ate1-deficient mice, observed during their initial loss of weight after TM treatment (Fig. 4A, B), was their lower core body temperature, on average 35.1°C, in comparison to identically TM-treated Ate1-containing control mice, whose average core body temperature was 36.0°C during the same time, in the absence of weight loss (Fig. 9E). After the early deaths of ∼15% of *Ate1^−/−^*;*CaggCreER* mice (Fig. 4A, B), the average temperature of surviving mice (36.6°C) was not significantly different from that of Ate1-containing control mice (36.7°C) (Fig. 9E). As one would expect from their depletion of WAT (Fig. 7A–C), Ate1-deficient mice were strongly hypersensitive to cold (Fig. 9F).

Ucp1 is a proton carrier in the mitochondrial inner membrane that mediates a partial uncoupling of oxidative phosphorylation from ATP synthesis, an alteration that can increase heat production and thereby regulate body temperature and energy homeostasis. Although Ucp1 is normally expressed in BAT but not in WAT, several mouse mutants other than *Ate1^−/−^* that are resistant to diet-induced obesity have been shown to ectopically express Ucp1 in WAT [32], [33]. Using RT-PCR and immunoblotting with anti-Ucp1 antibody, we found that the levels of *Ucp1* mRNA and Ucp1 protein in BAT did not change significantly between Ate1-deficient and Ate1-containing mice (Fig. 7G–I). Remarkably, however, the levels of both *Ucp1* mRNA and Ucp1 were strongly increased in WAT of Ate1-deficient mice (Fig. 7G–I). A Ucp1-Ate1 connection revealed by these findings adds a new dimension to the understanding of Ucp1 regulation ([32] and refs. therein), and may also provide an experimental route to identifying a relevant circuit that involves Ate1.

### Increased Metabolic Rate in Ate1-Deficient Mice

**Figure 10:**
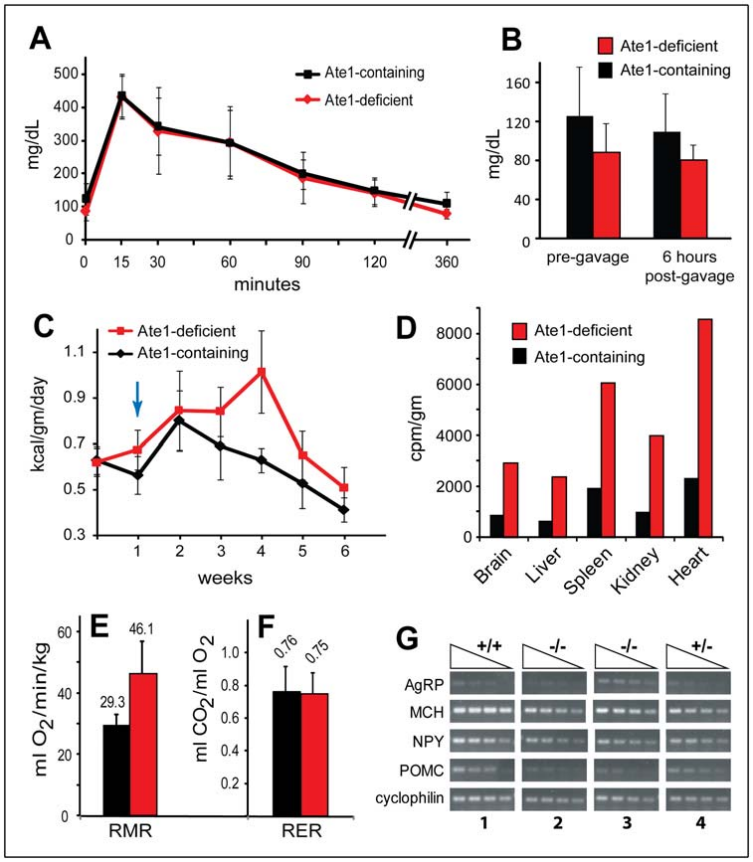
Energy balance and metabolic rate in Ate1-deficient mice. (A) Glucose tolerance test. Glucose concentration (mg/dL) in whole blood of Ate1-containing mice (n = 15; black curve) and Ate1-deficient mice (n = 11; red curve), at different times after a bolus of glucose by gavage, following a 16-hr fast. Glucose was administered at time zero. Error bars indicate ±SD. (B) Fasting blood glucose levels. Average blood glucose levels (mg/dL) in Ate1-containing mice (n = 15; black bar) and Ate1-deficient mice (n = 11; red bar), with measurements shortly before glucose gavage (after a 16-hr fast) and 6 hr after the gavage in A. Standard deviations are indicated. Statistical analysis was performed using an unpaired t-test (p<0.04). (C) Average daily energy consumption (kcal/gm of body weight) for Ate1-containing mice (n = 5; black curve) and Ate1-deficient mice (n = 3; red curve), with measurements from 1 week prior to tamoxifen (TM) treatment. Vertical arrow indicates the beginning of a 5-day TM treatment. Error bars indicate ±SD. (D) Relative efficiencies of the import of ^14^C-amino acids and ^14^C-peptides from gastrointestinal tract in Ate1-containing mice (black bars) versus Ate1-deficient mice (red bars). Shown here are representative comparisons of the retention of ^14^C (in cpm/gm) in the brains, livers, spleens, kidneys, and hearts of indicated mice 16 days after gavage with a single bolus of ^14^C-labeled proteins (see Materials and Methods). Mice were gavaged 26 days after TM treatment. (E) Comparison of resting metabolic rate (RMR) (measured in O_2_ (ml) consumed per kg of body weight per min) for Ate1-containing mice (n = 6; black bar) versus Ate1-deficient mice (n = 6; red bar). Standard deviations are indicated in E and F. Statistical analysis was performed using an unpaired t-test (p<0.008). (F) Comparison of the respiratory exchange ratio (RER), measured as CO_2_ (in ml) per ml of O_2_, for Ate1-containing mice (n = 6; black bar) and Ate1-deficient mice (n = 6; red bar) mice. No statistically significant difference in RER was observed. (G) RT-PCR analyses of *AgRP, MCH, HPY,* and *POMC* mRNA levels in the hypothalami of TM-treated Ate1-containing mice (Sets 1 and 4) versus Ate1-deficient mice (Sets 2 and 3). Set 1, *Ate1^flox/flox^* (+/+); Set 2, *Ate1^flox/flox^*;*CaggCreER* (−/−); Set 3, *Ate1^flox/flox^*;*CaggCreER* (−/−); Set 4, *Ate1^flox/+^*;*CaggCreER* (+/−). In sets 1 and 2, hypothalami were isolated 93 days after TM treatment. In sets 3 and 4 hypothalami were isolated ∼1 year after TM treatment. Sloping triangles indicate decreasing inputs (by 2-fold) of total RNA.

During the week prior to TM treatment, *Ate1^flox/−^*;*CaggCreER* and control (*Ate1^flox/+^*;*CaggCreER*) mice (at that point, both strains contained Ate1) consumed 0.63 and 0.62 kcal of standard chow per gram of body weight per day, respectively (Fig. 10C). Within a week after TM treatment the now Ate1-deficient *Ate1^−/−^*;*CaggCreER* mice increased their food consumption on average to 125% of identically TM-treated Ate1-containing mice (Fig. 10C). This pattern of significant hyperphagia of Ate1-deficient mice continued for the duration of this study, i.e., up to ∼8 months, with regular measurements for 6 weeks following TM treatment and intermittent comparisons afterwards (Fig. 10C). Thus, despite their initial decline of weight shortly after TM treatment and the early death of ∼15% of Ate1-deficient mice, and despite their subsequent failure to gain, on average, more than ∼63% and ∼69% of the weights of *Ate1* ^+/+^ and *Ate1* ^+/*−*^ mice, respectively, the Ate1-deficient mice consumed significantly more food than their Ate1-containing counterparts (Fig. 10C). To address their patterns of glucose utilization, we fasted these mice for 16 hr and measured blood glucose before after administering a 50-mg (0.2 ml) bolus of glucose by gavage. The kinetics of rise and fall of blood glucose levels under these conditions was similar for Ate1-deficient and Ate1-containing mice (Fig. 10A). Ate1-deficient mice had lower fasting glucose levels than Ate1-containing mice (88.6 mg/dl versus 125.3 mg/dl, respectively; p<0.04), and also lower glucose levels 6 hr after administration of glucose (80.9 mg/dl versus 109.7 mg/dl, respectively; p<0.04), consistent with the (expected) higher energy expenditure of Ate1-deficient mice, and suggesting normal sensitivity of these mice to insulin (Fig. 10B). There were no other significant differences in blood composition (as well as urine composition) between Ate1-containing and Ate1-deficient mice (Tables 2 and 3).

**Table 2:** Serum analyses.

	Ate1-deficient		Ate1-containing			
	Mean	StDev	Mean	StDev	Units	normal range
**Glucose**	153.3	41.9	182.6	73.3	**mg/dl**	62–175
**BUN**	26.0	3.8	22.0	8.0	**mg/dl**	12–28
**Creatinine**	0.4	0.1	0.4	0.1	**mg/dl**	0.3–1.0
**Sodium**	158.0	2.8	129.8	62.3		
**Potassium**	8.5	1.2	6.7	2.8		
**NA/K ratio**	18.8	2.4	17.1	7.2		
**Chloride**	112.8	3.4	91.9	43.4		
**CO2**	21.5	2.8	20.8	9.1		
**Anion gap**	32.3	1.9	24.3	11.6		
**Calcium**	9.3	0.4	8.1	3.4	**mg/dl**	3.2–8.5
**Phosphorus**	10.5	2.8	8.6	3.8	**mg/dl**	2.3–9.2
**Osm, Calc**	328.0	6.6	270.9	129.6		
**TP**	5.2	0.4	4.5	1.8	**g/dl**	3.5–7.2
**Albumin**	3.1	0.3	2.7	1.1	**g/dl**	2.5–4.8
**Globulin**	2.0	0.1	1.8	0.7	**g/dl**	0.6
**Albumin/Globulin**	1.5	0.1	1.3	0.5		4.1–8
**Bilirubin**	0.2	0.1	0.1	0.0	**mg/dl**	0.1–0.9
**AP**	102.3	41.6	113.7	48.0	**U/L**	∼70
**gamma gt**	0.0	0.0	0.0	0.0	**U/L**	
**ALT**	57.0	14.4	39.5	11.5	**U/L**	∼60
**AST**	409*	286.5	199.2	83.9	**U/L**	∼100
**Cholesterol**	70.1	4.0	71.5	31.8	**mg/dl**	26–82 (∼1.5 mmol/L)
**T4**	2.7	0.9	2.4	0.8	**ng/dl**	
**T3**	54.3	7.4	50.9	19.7	**µg/dl**	

* Ate1-deficient mice with more severe phenotypes tended to have higher AST levels.

**Table 3:** Urinalysis.

-	Ate1-deficient	Ate1-containing	Units
**Glucose**	neg	neg	mg/dL
**Bilirubin**	neg/small	neg/small	
**Ketone**	neg	neg	mg/dL
**Specific Gravity**	1.03	1.02	
**Blood**	neg	neg	
**pH**	6.2*	7.1	
**Protein**	100	100	mg/dL
**Urobilinogen**	0.2	0.2	mg/dL
**Nitrite**	neg	neg	
**Leukocytes**	neg	neg	

* Ate1-deficient mice had a significantly lower urine pH.

To measure metabolic rate, we employed indirect calorimetry (see Materials and Methods), determining O_2_ consumption and CO_2_ production by mice under resting conditions. The metabolic rate (resting metabolic rate, RMR) of Ate1-deficient mice was indeed higher than normal: they consumed on average 46.12 ml of O_2_ per kg per min, versus 29.3 ml of O_2_ per kg per min for Ate1-containing mice (Fig. 10E). In contrast, the respiratory exchange ratio, RER (the ratio of CO_2_ eliminated from the lungs to O_2_ taken into the lungs), a parameter that depends on a preferred source of fuel (e.g., carbohydrates versus fat), was similar for Ate1-deficient and Ate1-containing mice: 0.75 and 0.76, respectively (Fig. 10F).

The S. cerevisiae N-end rule pathway regulates the import of short peptides through the conditional degradation of Cup9, the import's repressor [34]. It is likely (but remains to be verified) that the N-end rule pathway regulates the transmembrane traffic of peptides in mammals as well. To address the possibility that significantly lower weights (despite hyperphagia) of Ate1-deficient mice might stem, at least in part, from an impaired ability to import peptides and/or amino acids from their gastrointestinal (GI) tract, we labeled E. coli with a mixture of ^14^C-amino acids and isolated a ^14^C-protein fraction that was essentially free of nucleic acids, fatty acids, lipids and carbohydrates (see Materials and Methods). Ate1-deficient and Ate1-containing mice were gavaged with a bolus of this ^14^C-protein preparation, followed by measurements of ^14^C in several organs of these mice (and in their feces) as a function of time post-gavage. Ate1-deficient mice passed less ^14^C in feces than Ate1-containing mice (Fig. 9D). Moreover, Ate1-deficient mice accumulated more of ^14^C in their brains, livers, spleens, kidneys and hearts than Ate1-containing mice (Figs. 9C and 10D). Irrespective of mechanistic causes involved (they remain to be understood), higher than wild-type levels of protein-derived ^14^C delivered to tissues of Ate1-deficient mice indicated the absence of significant defects in their transport of peptides and/or amino acids from GI tract.

Given the metabolic and behavioral abnormalities of Ate1-deficient mice (Figs. 6C–E, 8A, D and 10E, F), we also examined them for expression of neuropeptides. As we would be interested, at present, only in strong differences, a semiquantitative RT-PCR was employed. Using total RNA from hypothalami of Ate1-deficient versus Ate1-containing mice, we found no consistent differences between these mice in regard to the levels of hypothalamic mRNAs that encoded the agouti-related protein (AgRP) and the neural peptide Y (NPY) (Fig. 10G). Strikingly, however, there was a consistent and strong decrease of expression, in Ate1-deficient mice, of mRNA encoding proopiomelanocortin (POMC) (Fig. 10G). POMC is a precursor of several neurohormones with broad systemic and brain-specific functions ([35] and refs. therein). These functions include a role in melanocyte regulation (a process that is likely to be perturbed in Ate1-deficient mice; see above) and a down-regulation of food intake (the observed deficiency in POMC is consistent with hyperphagia of Ate1-deficient mice (Fig. 10C, G)). Similarly to a connection between Ate1 and the Ucp1 uncoupling protein (Fig. 7G–I), our finding of a link between N-terminal arginylation and the expression of POMC is likely to provide an experimental route to identifying the relevant Ate1-dependent circuit.

## Discussion

A cell is alive owing to a cell-wide dynamic network of structurally or functionally interacting biopolymers. Some parts of this network can be sufficiently insulated, through their design, to be considered, in the first approximation, as distinct circuits. The N-end rule pathway is one such circuit. Its enzymes receive as their input specific degron-bearing proteins and convert them, through deamidation, arginylation, polyubiquitylation and processive degradation, into an output of proteolysis-derived short peptides (Fig. 1A). The rate and selectivity of the proteasome-mediated protein degradation by the N-end rule pathway are modulated by physiological effectors, including specific phosphokinases, short peptides, redox, heme and nitric oxide (see Introduction). Some of N-end rule substrates are produced by proteases that include MetAPs, separases, caspases and calpains. These and other nonprocessive proteases, which function as upstream components of the N-end rule pathway, have in common their ability to convert, through a cleavage, a pro-N-degron into an N-degron.

The present study expanded the earlier understanding of the Ate1 R-transferase (Fig. 1A) by making possible a postnatal inactivation of mouse *Ate1*. (Unconditional deletion of *Ate1* results in embryonic lethality [10].) Described and discussed in Results is a large set of defects, some of them quite striking, in juvenile and adult mice upon the postnatal inactivation of *Ate1* and the resulting loss of N-terminal arginylation (Figs. 1C, D). The initial abnormality is a rapid decrease of body weight and early death of ∼15% of Ate1-deficient mice, with surviving mice attaining, gradually, only ∼70% of the weight of wild-type mice identically treated with tamoxifen (TM) (Fig. 4A–C). Both “partial” lethality and the transiency of acute crisis, over ∼3 weeks after TM treatment (red arrow in Fig. 4B), remain to be understood in molecular terms. This crisis and subsequent failure to thrive occur despite higher than normal food intake by Ate1-deficient mice (Fig. 10C). These mice contain little or no visceral fat (Figs. 5D and 7A–E), and exhibit an increased metabolic rate (Fig. 10E), resistance to diet-induced obesity (Fig. 7F), enlarged brains (Figs. 5D and 6A), kyphosis (Fig. 5B), a striking hyperkinesia (Figs. 6C–E and 8A), and male sterility (Fig. 6F–K).

Owing to current constraints of the Cre-lox technology ([25] and refs. therein), the extent of *Ate1* inactivation, while nearly 100% in some mouse tissues, was variable in others. The TM-induced *Ate1^−/−^*;*CaggCreER* mouse strains are thus mosaics of *Ate1^flox/−^* and *Ate1^−/−^* cells, where *Ate1^−/−^* cells are a great majority in most organs, such as the brain, but even there do not reach 100% of all cells (see Results). The initial weight loss upon the TM-induced conversion of *Ate1^flox/−^* mice to *Ate1^−/−^* mice was accompanied by death of ∼15% of *Ate1^−/−^* mice (Fig. 4A, B). Such a “partially” lethal phenotype suggests that an adult-onset *Ate1^−/−^* genotype in all cells (as distinguished from most cells) of a mouse might be incompatible with viability, similarly to the embryonic lethality of unconditional *Ate1^−/−^* mice. Thus, paradoxically, the discovery, in the present study, of specific Ate1-linked defects in adult mice might have been made possible by incomplete penetrance of the Cre-induced conversion of *Ate1^flox^* to the *Ate1^−^* allele.

The set of definitively identified mammalian N-end rule substrates that involve N-terminal arginylation consists, at present, of fewer than 10 proteins. They subserve different functions, from chromosome segregation to control of apoptosis and regulation of G proteins (see Introduction). This set is the tip of the iceberg, as several considerations [2], in addition to our findings above, strongly suggest a larger number of physiological Ate1 substrates. Given this complexity, the specific and often striking phenotypes of Ate1-deficient mice that were discovered in the present work will be of major assistance in deciphering the underlying Ate1 circuits.

## Methods

### Animal Care and Treatments

All animal care and procedures in the present study were conducted according to the relevant NIH guidelines, and were approved (Protocol #1328) by the Institutional Animal Care and Use Committee, the Office of Laboratory Animal Research (OLAR) at the California Institute of Technology, where the entire present study was carried out. Mice were housed at ∼22°C, at a pathogen-free (barrier) facility, using a 12 hr light/12 hr dark cycle, with Laboratory Rodent Diet 5001 (PMI International, Richmond, IN) ad libitum. Mice aged between 3 and 8 weeks were treated with tamoxifen (TM) (Sigma) (2 mg in 0.2 ml sesame oil) by daily intraperitoneal (IP) injections over 5 days. Mice were weighed weekly, starting 3 days before the first TM treatment. For a high-fat diet (HFD) study, mice were fed ad libitum a diet containing 35.5% fat (BioServe, Frenchtown, NJ), and were weighed weekly.

### Construction of *Ate1^flox/−^*;*CaggCreER* Mouse Strains

Mouse genomic DNA encoding Ate1 was isolated from a BAC library [10]. Two pBluescript-based plasmids were used to construct the targeting vector. In one insert, a ∼12 kb HindIII fragment contained *Ate1* exons 1a, 1b, 2, and 3 as well as ∼1.9 kb of DNA 3′ of exon 3 (ending just before exon 4). In the other insert, a ∼2.9 kb fragment contained *Ate1* exons 4 and 5. The entire ∼12 kb HindIII fragment and a part of the ∼2.9 kb fragment were modified as described below and assembled into a final ∼22.5 kb targeting vector consisting of the following parts (Fig. 2C): (**i**) pBR322 backbone (New England Biolabs, Ipswich, MA); (**ii**) a ∼6.3 kb “long arm” of *Ate1* homology containing the *Ate1* exon 1a, the bidirectional P*_Ate1_* promoter [14], and exon 1b; (**iii**) A single *loxP* site ∼300 bp upstream of *Ate1* exon 2; (**iv**) a ∼2 kb fragment that contains, 50 bp downstream of Ate1 exon 4, a “floxed” *Hph* (hygromycin) antibiotic-resistance marker, expressed from the P*_PGK_* promoter [36]; (**v**) a ∼1.2 kb “short arm” of *Ate1* homology that spans most of the intron between exons 4 and 5; (**vi**) a gene encoding HSV-TK (herpes simplex virus thymidine kinase), expressed from the P*_PGK_* promoter. The targeting vector was linearized with BamHI and electroporated into CJ7 embryonic stem (ES) cells (a gift from Dr. Thomas Gridley, formerly of Jackson Laboratories, Bar Harbor, ME). ES cells were grown in DMEM supplemented with 15% fetal bovine serum (FBS), 0.1 mM non-essential amino acids, 0.1 mM β-mercaptoethanol, 2 mM glutamine, 100 U/ml penicillin, 0.1 mg/ml streptomycin, 1 mM pyruvate, and 1,000 U/ml leukemia inhibitory factor (LIF) [37], using a feeder layer of hygromycin-resistant mouse primary fibroblasts that had been treated with 10 µg/ml mitomycin C for 3 hr at 37°C. Selection with hygromycin (at 0.2 mg/ml) and 1-(2′-deoxy, 2′-fluoro-β-D-arabinofuranosyl)-5-iodouracil (FIAU; at 0.4 µM) was started 24 hr after electroporation. Correctly targeted ES cell clones that contained “tri-loxed” *Ate1* allele (Figs. 2C, D) were identified using Southern hybridization and PCR. Southern DNA probes and positions of primers for PCR are indicated in Fig. 2.

Two correctly targeted, independently produced ES cell lines that had apparently normal karyotypes were injected into 3.5-days-postcoitum C57BL/6J blastocysts and implanted into pseudopregnant females. The resulting male chimeric offspring were mated with C57BL/6J females. In some of the progeny, “floxed” ES cells became a part of germ line. Standard mating techniques [36], [38] were then used to produce, initially, mouse strains that contained a “tri-lox” *Ate1* configuration, in that they also contained the floxed positive-selection P*_PGK_*-*hph* cassette (Fig. 2D). This DNA segment was removed by mating *Ate1*-tri-lox heterozygotes with EIIa-Cre mice that expressed Cre recombinase only in early, pre-implantation blastocysts [39], [40], [41]. Owing to the presence of three *loxP* sites at the initial floxed *Ate1* locus, F1 progeny from this cross were mosaic, i.e., their tissues, including germ line, contained varying configurations of retained *loxP* sites, depending on specific patterns of Cre-mediated recombination (Fig. 2A–F). To isolate a mouse strain with the desired configuration of (retained) *loxP* sites (Fig. 2E), the above F1 mosaic mice were mated to wild-type C57BL/6 mice. This produced, among other progeny, a strain that lacked the P*_PGK_*-*Hph* cassette and had the desired *Ate1^flox/+^* genotype, in the (mixed) C75BL/6J-129SvEv background.

Through the use of appropriate mating pairs, with genotyping of resulting progeny, we produced *Ate1^flox/−^*;*CaggCreER* mice as well as *Ate1^flox/flox^*;*CaggCreER* mice (Fig. 2). The former strain harbored one unconditionally null *Ate1^−^* allele (derived, through matings, from the previously constructed unconditional heterozygous *Ate1^+/−^* mice [10]) and one “floxed”, conditionally active *Ate1^flox^* allele that could be made null in the presence of active Cre recombinase. In the latter strain (*Ate1^flox/flox^*;*CaggCreER*), both copies of *Ate1* were *Ate1^flox^*. These mouse strains also contained the *CaggCreER* gene, expressed from the ubiquitously active chimeric *Cagg* promoter (Fig. 2E) [25]. *CaggCreER* encoded CreER, a fusion between the phage P1 Cre recombinase and a derivative of the mouse estrogen receptor ligand binding domain. CreER was functionally inactive (sequestered in the cytosol) but could be activated by intraperitoneal (IP) injection of tamoxifen (TM) [25]. To produce *Ate1^flox/−^*;*CaggCreER* mice, we mated *Ate1^flox/+^* mice with *Ate1^+/−^*;*CaggCreER* mice (the latter were generated by mating *Ate1^+/−^* with *Ate1^+/+^*;*CaggCreER* mice). To produce *Ate1^flox/flox^*;*CaggCreER* mice, we mated *Ate1^flox/+^* mice with *Ate1^flox/+^*;*CaggCreER* mice (the latter were generated by mating *Ate1^flox/+^* mice with *Ate1^+/+^*;*CaggCreER* mice). In the notations here and elsewhere in the paper, “flox-on” indicates a configuration depicted in Fig. 2E (the functionally active *Ate1^flox^* allele), whereas “flox-off” indicates a configuration depicted in Fig. 2F (the null *Ate1^−^* allele).

### Southern Hybridization and PCR

Total genomic DNA was isolated from ES cells by washing them twice with phosphate-buffered saline PBS, followed by an overnight incubation at 50°C in 10 mM EDTA, 10 mM NaCl, 0.5% Sarcosyl,10 mM Tris-HCl (pH 7.5) containing Proteinase K at 0.2 mg/ml. Thereafter an equal volume of 75 mM NaCl in 100% ethanol was added. Precipitated genomic DNA was then gently washed twice with 70% ethanol and resuspended in T_10_E_0.1_ buffer (10 mM Tris (pH 8.0), 0.1 mM EDTA). Total genomic DNA was isolated from mouse tails or other tissues by overnight incubation at 55°C, with constant rotation, in 5 mM EDTA, 0.2 M NaCl, 0.3% SDS, 0.1 M Tris (pH 8.5) containing Proteinase K at 0.4 mg/ml. Thereafter an equal volume of isopropanol was added, and the mixture was gently inverted several times. Genomic DNA was then precipitated, and washed twice, with 70% ethanol, followed by a gentle resuspension in T_10_E_0.1_ buffer.

Southern hybridization was performed as described [42], with a ^32^P-labeled mouse DNA probes that was produced by PCR using the following primers: CB108F and CB107R (Table 2) to amplify a 219 bp genomic fragment containing *Ate1* exon 5 (probe D, external probe); CB23 and CB24 (Table 4) to amplify a 929 bp genomic fragment that was a part of the long arm of the targeting vector (Probe A, internal probe) (Fig. 2). DNA probes were labeled with ^32^P-labeled using the Rediprime-II Random Prime Labeling System (Amersham Biosciences, Piscataway, NJ) according to the manufacturer's protocol. Hybridization with Probe A was carried out overnight at 57°C in ExpressHyb solution (Clontech). The membrane was then washed once for 10 min at room temperature (RT) in 2xSSC/0.1% SDS, once for 30 min at 55°C in 2xSSC/0.1% SDS, once for 30 minutes at 58°C in 0.5xSSC/0.1% SDS, and once for 30 minutes at 65°C in 0.1xSSC/0.1% SDS, followed by autoradiography. (1xSSC is 0.15 M NaCl, 15 mM Na-citrate, pH 7.4.) Hybridization with Probe D was carried out overnight at 55°C in ExpressHyb solution (Clontech). The membrane was then washed once for 10 min at RT in 2xSSC/0.1% SDS, once for 30 min at 55°C in 2xSSC/0.1% SDS, and once for 30 min at 58°C in 1xSSC/0.1% SDS, followed by autoradiography.

**Table 4:** PCR primers used in the present study.

Name	Nucleotide sequence (5′ to 3′)	Use
CB23	ACTTTACAGTTGCTAGATAAGC	for PCR of Southern Probe A
CB24	AGCAGGTTACTTGTCCAGTC	for PCR of Southern Probe A
CB107R	AATTCTTTAGACCCTTCTTTGTTT	for PCR of Southern Probe D
CB108F	TGTCAATAATGCAGCTGATGATGGGCTTTCATTGTCTTCTCATTCTTAGATGAGCCCATGGATTCTAC	for PCR of Southern Probe D
CB156F	CAA GCAG GGG AAG GAG GC	PCR detection ATE1-floxON
CB157R	TTC AGG AGT TAG CCA TTG CC	PCR detection ATE1-floxON and ATE1-floxOFF
AK49	GGT ATT TGC TGC CGT CCT TTG GTG GT	PCR detection of ATE1-null
YT641	CTG TTC CAC ATA CAC TTC ATT CTC AG	PCR detection of ATE1-null
AK83-Cbfix	CTG GAG ACA AAG CCC CAG CCA GAC	PCR detection of ATE1-null
Cre-1	GTT CGC AAG AAC CTG ATG GAC A	PCR detection of Cre gene
Cre-2	CTA GAG CCT GTT TTG CAC GTT C	PCR detection of Cre gene
CB159R	AC TGT AGA ATC CAT GGG CTC	PCR detection wild type ATE1
CB160F	ACA GCA TAA GTG AGA CAC TCA	PCR detection wild type ATE1
CB110F	GTT TGT GTC ACC ACT CCT ACC	PCR detection ATE1-floxOFF
oIMR0042	CTA GGC CAC AGA ATT GAA AGA TCT	PCR detection IL-2 control
oIMR0043	GTA GGT GGA AAT TCT AGC ATC ATC C	PCR detection IL-2 control
AgRP-for	GCGGAGGTGCTAGATCCA	RT-PCR
AgRP-rev	AGGACTCGTGCAGCCTTA	RT-PCR
NPY-for	CTCCGCTCTGCGACACTAC	RT-PCR
NPY-rev	AATCAGTGTCTCAGGGCT	RT-PCR
POMC-for	ACCTCACCACGGAGAGCA	RT-PCR
POMC-rev	GCGAGAGGTCGAGTTTGC	RT-PCR
MCH-for	ATTCAAAGAACACAGGCTCCAAAC	RT-PCR
MCH-rev	CGGATCCTTTCAGAGCAAGGTA	RT-PCR
cyclophilin-for	GGTGGAGAGCACCAAGACAGA	RT-PCR
Cyclophilin-rev	GCCGGAGTCGACAATGATG	RT-PCR
Ucp2-5′	GGGGCGGCCGCATGGTTGGTTTCAAGGCCAC	RT-PCR
Ucp2-3′	GGGGCGGCCGCTCAGAAAGGTGCCTCCCGAG	RT-PCR
Actb-5′	ATGGATGACGATATCGCTGCG	RT-PCR
Actb-3′	GAAGCTGTAGCCACGCTCGG	RT-PCR
Leptin-5′	GGGGCGGCCGCATGTGCTGGAGACCCCTGTG	RT-PCR
Leptin-3′	GGGGCGGCCGCTCAGCATTCAGGGCTAACAT	RT-PCR
Ucp1-5′	GGGGCGGCCGCATGGTGAACCCGACAACTTC	RT-PCR
Ucp1-3′	GGGGCGGCCGCTTATGTGGTACAATCCACTG	RT-PCR

### Genotyping of Mouse Strains

PCR-based genotyping was carried out with total genomic DNA isolated from various mouse tissues. Routine genotyping was performed using DNA from mouse tails. Specific *Ate1* alleles and transgenes encoding specific derivatives of Cre were identified as follows. A two-primer PCR using the CB156F and CB157R primers (Table 4) was employed to produce and detect a 512 bp fragment of the *Ate1^flox^* (“floxON”, active) allele as well as a 472 bp fragment of the wild-type *Ate1^+^* allele. A four-primer PCR using the CB110F, CB157R, OIMR0042, and OIM0043 primers Z (Table 4) was employed to produce and detect a 470 bp fragment of the *Ate1^flox^*-derived *Ate1^−^* allele (“floxOFF”) as well as a (control) 324 bp fragment of the *Il-2* gene. A three-primer PCR using the AK49, YT641, and AK83-CBfix (Table 4) was employed to detect both a 300 bp fragment of the unconditional *Ate1^−^* allele [10] and a 560-bp fragment of the wild-type *Ate1^+^* allele. A four-primer PCR using the Cre-1, Cre-2, CB159R, and CB160F primers (Table 4) was employed to detect both a 320 bp fragment of the *CaggCreER* transgene as well as a 1,060 bp fragment of the wild-type *Ate1^+^* allele. All PCR reactions except for those to detect the *CaggCreER* transgene were carried out using HotStar Taq DNA polymerase, standard buffer conditions (Qiagen, Valencia, CA), 35 cycles of template denaturation for 30 seconds at 95°C, followed by primer annealing for 30 seconds at 60°C and primer extension for 1 minute at 72°C. PCR reactions for detecting *CaggCreER* were carried out using 30 cycles of template denaturation for 30 seconds at 95°C, followed by primer annealing for 30 seconds at 58°C and primer extension for 45 seconds at 72°C.

### Northern and RT-PCR Analyses of RNA

Total RNA was isolated from various mouse tissues using the RNeasy Protect Mini Kit (Qiagen). Tissue disruption and homogenization were done in Buffer RLT and the MP FastPrep-24 instrument with Lysing Matrix D (MP Biomedicals, Solon, OH) for 2 runs at 6.5 m/s. First-strand cDNA was primed with oligo-dT using the SuperScript III First-Strand Synthesis System (Invitrogen, Carlsbad, CA) and PCR was carried out using primers cited in legends to the corresponding figures and in Table 4.

### Tissue Extracts and Immunoblotting

Various mouse tissues were harvested and lysed in “Tissue Lysis Buffer” (10% glycerol, 0.05% NP40, 0.15 M NaCl, 2 mM EDTA, 1 mM dithiothreitol (DTT) 1 mM phenylmethylsulfonyl fluoride PMSF 50 mM HEPES, pH 7.5) plus freshly dissolved “Complete EDTA-Free Protease Inhibitors” (Roche), using the MP FastPrep-24 instrument and Lysing Matrix D (MP Biomedicals, with 2 or 3 runs at 6.5 m/s for 25 sec each, and with 5-min incubations on ice between the runs. The lysates were centrifuged at 10,000*g* for 20 min at 4°C. The supernatants were fractionated by SDS-12.5% PAGE, transferred to Immobilon-P PVDF membranes (Millipore, Billerica, MA), and analyzed by immunoblotting (IB) with antibodies indicated in specific figures. Immunoblots were visualized using SuperSignal West Pico or SuperSignal West Dura reagents (Thermo Scientific, Rockford, IL) according to the manufacturer's instructions.

### Other Analyses of Mouse Tissues

Specific mouse tissues were dissected immediately after euthanasia by CO_2_ inhalation. The tissues washed with PBS, blotted dry on Kimwipes, and weighed (wet). For dry-weight measurements, mouse brains were dissected intact, washed in PBS, blotted dry on Kimwipes, weighed wet, then incubated overnight in acetone. After acetone incubation, individual brains were lyophilized until their (dry) weight no longer decreased.

For routine histological examinations, tissues or organs were fixed in Bouin's solution or in 4% formaldehyde, using standard procedures [37]. Fixed samples were embedded in paraffin, sectioned, and stained with hematoxylin and eosin. To stain for LacZ (NLS-βgal), dissected tissues or organs were fixed in LacZfix (0.2% glutaraldehyde, 5 mM EGTA, 0.1 M MgCl_2_ in PBS (pH 7.3)) for 4 hr, rinsed twice with PBS, dehydrated overnight at 4°C in 30% sucrose, 2 mM MgCl_2_ in PBS, and embedded and frozen in Tissue-Tek O.C.T. Compound (Sakura Finetek USA, Inc. Torrance, CA). Cryosections (prepared using a Tissue Tek Microtome/Cryostat model 4553) were mounted onto glass slides, fixed in LacZfix for 10 min at RT, washed 3 times in LacZWash (0.02% NP40, 01% Na-deoxycholate, 2 mM MgCl_2_ in PBS), and stained overnight at 37°C with LacZ stain (LacZWash containing 1 mg/ml XGal, 5 mM K_4_Fe(CN)_6_ and 5 mM K_3_Fe(CN)_6_). Stained sections were washed with PBS and mounted with Permount for light microscopy. Apoptosis was assessed by TUNEL, a nuclear DNA fragmentation assay, using a TUNEL kit (Roche, Indianapolis, IN), fluorescein-dUTP, and manufacturer's instructions. Cell proliferation was assayed using the Click-It Edu Cell Proliferation kit (Invitrogen).

### In Vitro Arginylation Assay

The arginyl-transferase (R-transferase) reaction (50 µl) contained extracts for a specific mouse tissue (2.5 mg of total protein per ml), α-lactalbumin (arginylation reporter [14]) (0.5 mg/ml), total E. coli tRNA (0.6 mg/ml) (Sigma), total E. coli aminoacyl-tRNA synthetases (800 U/ml) (Sigma), 5 mM MG132 (proteasome inhibitor) (Sigma), 1 mM ATP, 30 mM KCl, 2 mM MgCl_2_, 2 mM β-mercaptoethanol, 10 mM Tris-HCl (pH 8.0) and 0.3 mM ^3^H-arginine (PerkinElmer, NEN Radiochemicals, Waltham, MA). The reaction mixture was incubated for 30 min at 37°C and deposited onto GF/C filter disks (GE-Healthcare, Pittsburg, PA). The filters were thereafter incubated for 10 min in 10% cold CCl_3_COOH, followed by 10 min in 5% CCl_3_COOH at 95°C. The filters were then washed in 5% CCl_3_COOH 3 times at RT, followed by a single ether∶ethanol (1∶1) wash, two ether washes, and measurements of ^3^H retained on a filter using a scintillation spectrometer.

### Blood and Urine Analyses

Blood (∼0.6 ml per mouse) was withdrawn by cardiac puncture and transferred into BD Microtainer SST tubes (BD, Franklin Lakes, NJ). The serum fraction was prepared by centrifugation in a microcentrifuge after clotting occurred, immediately frozen in liquid N_2_ and stored at −80°C. The levels of glucose, cholesterol, sodium, potassium, chloride, calcium, phosphorus, blood urea nitrogen, creatine, total protein, albumin, total bilirubin, aspartate aminotransferase (AST), alanine aminotransferase (ALT), alkaline phosphatase, γ-glutamyltransferase (gamma gt), as well as the T3 and T4 hormones were determined by Phoenix Central Laboratories (Everett, WA).

Urine was obtained by placing the external urethra over a test tube. Urine samples collected from Ate1-deficient mice were pooled and compared with pooled urine from Ate1-containing mice. The levels of glucose, bilirubin, ketones, blood, protein, urobilinogen, nitrite, leukocytes, as well as the pH and specific gravity were determined using the Multistix 10 SG Reagent Strips (Bayer, Tarry Town, NY).

### Measurements of Body Temperature and Cold Sensitivity

Mice (housed at one mouse per cage, without bedding but with food and water) were exposed to a 4°C environment for up to 6 hr Their core body temperature was monitored every 30 min via a rectal probe digital thermometer (Thermalert TH-8; Physitemp Instruments., Clifton, NJ).

### Measurements of ^14^C-Protein Uptake from Gastrointestinal Tract

E. coli DH5α cells were grown in minimal M9 media supplemented with 0.5% glucose and 50 µCi of ^14^C-amino acids (derived from ^14^C-protein hydrolysate (Amersham)) until the incorporation of ∼70% of the added ^14^C amino acids. Cells were lysed by one freeze/thaw cycle in PBS containing 1 mg/ml lysozyme and incubated with RNase H and DNase I for 45 min at 37°C. A crude protein fraction was isolated by precipitation with cold 10% TCA (CCl_3_COOH), and the pellet was washed with ice-cold acetone. The pellet was redissolved in PBS, with a brief sonication to facilitate solubilization. 0.2 ml of the resulting sample, containing 260,000 cpm of ^14^C-labeled E. coli proteins was fed to a mouse by oral gavage. Urine and feces was collected at various times post-gavage. Total ^14^C was measured, using a scintillation spectrometer, in feces, urine, and (eventually) in mouse tissue samples that were collected either 48 hr or 15 days post-gavage.

### Measurements of Glucose Uptake

For glucose analyses, mice were fasted for 24 hr, then gavaged with 50 mg glucose in 0.2 ml of water. Blood was collected through the lateral tail vein at 15, 30, 60, 90, 120, and 360 min post-gavage. Blood glucose levels were determined using the OneTouch UltraMini Blood Glucose Monitoring System (LifeScan, Johnson and Johnson, Milpitas, CA).

### Metabolic Analyses

The resting metabolic rate was determined at the Mouse Physiology Laboratory in the Department of Physiology at the Geffen School of Medicine, UCLA using indirect calorimetry as previously described [43], [44]. Single mice were placed into a custom-made enclosed plexiglas chamber (25 cm×12 cm×7.5 cm, with 4 room air intake vents and one outflow port) and allowed to come to rest over a period of 30 min to 2 hr. Outflow of expired gases was sampled by the gas analyzer and recorded using a computerized acquisition system during a 30-min resting interval.

### MRI Analyses of Mouse Brains

The procedures used were essentially the same as previously described [45]. Mice were given an IP injection of 40 mg/kg of Na-pentobarbital (Nembutal, Hospira, Inc., Lake Forest, IL). Once fully anesthetized, mice were transcardially perfused with 4% formaldehyde in PBS. MRI analysis was performed by the Caltech Brain Imaging Center. Briefly, mouse heads were excised and postfixed in 4% formaldehyde/PBS overnight. Hair and skin were removed from fixed heads, which were then soaked in 5 mM Gadolinium-based MR contrast agent (Prohance Bracco Diagnostics, Durham, NC) for 10 days, to decrease the intrinsic tissue relaxation rates and improve the MR acquisition efficiency. A gradient echo sequence (TE/TR = 8 msec/50 msec, 16 averages) was used to acquire 3D data sets of the mice heads, using a Bruker 7T Biospec animal magnet system. Images were reconstructed with an isotropic resolution of ∼90 µm and analyzed using Brainsuite 2 software [46].

### Open-Field and Startle Response Tests

For the open-field activity measurements, individual mice were placed into a square chamber (50 by 50 cm). Movements along the *x* and *y* axes were tracked and analyzed using Ethovision software (Noldus, Leesburg, VA) over 15-min intervals. Startle response tests were carried out essentially as described previously [47].
